# Supplementary material for: Multiregional transcriptomics identifies congruent consensus subtypes with prognostic value beyond tumor heterogeneity of colorectal cancer
Source: Nat Commun. 2024 May 21;15:4342. doi: 10.1038/s41467-024-48706-2 (PMC11109119; doi:10.1038/s41467-024-48706-2)
Supplement: Supplementary file 1 — Supplementary Information [file 41467_2024_48706_MOESM1_ESM.pdf]

Supplementary Information for: “Multiregional transcriptomics identifies congruent consensus subtypes with prognostic value beyond tumor heterogeneity of colorectal cancer”

Jonas Langerud, Ina A. Eilertsen, Seyed H. Moosavi, Solveig M. K. Klokkerud, Henrik M. Reims, Ingeborg F. Backe, Merete Hektoen, Ole H. Sjo, Marine Jeanmougin, Sabine Tejpar, Arild Nesbakken, Ragnhild A. Lothe, Anita Sveen

Table of Contents

Supplementary Figure 1. Overview of sample sets and gene expression platforms 3

Supplementary Figure 2. Tumor size, sample number, general transcriptomic heterogeneity and histological cryosections of multiregional samples 4

Supplementary Figure 3. Gene set enrichment analyses of heterogeneous versus homogeneous tumors for each CMS group in the multiregional sample set 6

Supplementary Figure 4. Differentially expressed genes between tumors with heterogeneous versus homogeneous CMS classifications in the multiregional sample set 7

Supplementary Figure 5. Abundance of cancer associated fibroblasts in tumors with homogeneous and heterogeneous CMS classifications of multiregional samples 8

Supplementary Figure 6. Overview of computational approach for estimation of intra-tumor CMS heterogeneity in primary CRCs with a single sample 9

Supplementary Figure 7. Concordance of CMS classifications according to the original classifier and singscore enrichments in the single-sample primary tumor set 10

Supplementary Figure 8. Evaluation of computational approach for classification of intra-tumor CMS heterogeneity of single samples 11

Supplementary Figure 9. Five-year relapse-free survival according to intra-tumor CMS heterogeneity....12

Supplementary Figure 10. Limited concordance in intra-tumor heterogeneity of CMS and CRIS classifications 13

Supplementary Figure 11. Intra-tumor versus inter-sample gene expression variation in the multiregional sample set 14

Supplementary Figure 12. Distribution of genes according to ITH-score 15

Supplementary Figure 13. Gene set correlations of ITH-intermediate genes 16

Supplementary Figure 14. Gene set overrepresentation analysis of ITH-low cancer-critical genes 17

Supplementary Figure 15. Validation of ITH-high and ITH-low gene categories in single-cell RNA sequencing data .18

Supplementary Figure 16. Gene set enrichment analyses of subtypes in the ITH-low k2 and iCMS frameworks 19

Supplementary Figure 17. Principal components analysis of primary CRC and liver metastasis samples 20

Supplementary Figure 18. Cophenetic and silhouette scores from NMF clustering of different sample sets with a predefined interval of factorization ranks 21

Supplementary Figure 19. Alluvial plots of concordance in NMF classification between sample sets 22

Supplementary Figure 20. Gene set enrichment analyses of subtypes based on ITH-low genes 23

Supplementary Figure 21. NMF classification based on ITH-low genes defined by previously published threshold .24

Supplementary Figure 22. Classification concordance between iCMS and cCMS 25

Supplementary Figure 23. Differential expression of ITH-low genes among sample clusters split from the k2 cluster corresponding to iCMS3 26
Supplementary Figure 24. PCA plot comparing the CMS and cCMS classification frameworks....28
Supplementary Figure 25. Gene set enrichment analyses according to cCMS and CMS classifications 29
Supplementary Figure 26. Classification concordance from subtype discovery of all primary CRC samples versus MSS samples only 30
Supplementary Figure 27. Patient survival according to cCMS 31
Supplementary Figure 28. Kaplan-Meier plot of five-year relapse-free survival according to CMS in stage I-III CRC (in the subgroup of patients with concordant intra-tumor cCMS classifications)....32
Supplementary Figure 29. Classification of the GSE39582 series (*n*=566 primary tumors) based on ITH-low genes .33
Supplementary Figure 30. Classification of the TCGA series (*n*=573 primary tumors) based on ITH-low genes 35
Supplementary Figure 31. The posterior probability threshold has a limited effect on the frequency of intra-tumor CMS heterogeneity....36
Supplementary Figure 32. Comparisons of CMS scores from different methods in the multiregional sample set 37
Supplementary Figure 33. Gene set correlations for ITH-low genes according to original and custom ITH-score thresholds....38

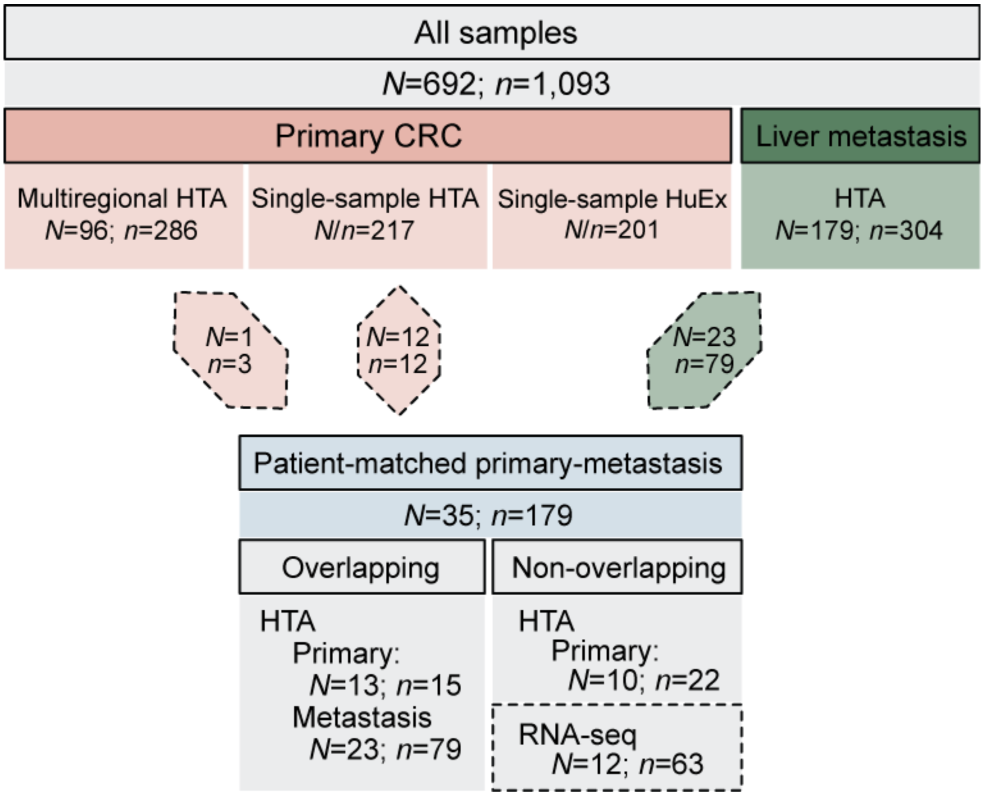

Supplementary Figure 1. Overview of sample sets and gene expression platforms

Overview of the in-house sample sets analyzed in the study, indicating sample type (primary CRC or liver metastasis) and gene expression platform (HTA or HuEx microarrays or RNA sequencing, RNA-seq). Upper case *N* indicates the number of patients and lower case *n* the number of samples. Diamond-shaped arrows indicate samples included for patient-matched primary-metastasis comparisons (all overlapping samples were analyzed on HTA), while the remaining matched samples (HTA or RNAseq) were included only for longitudinal comparisons.

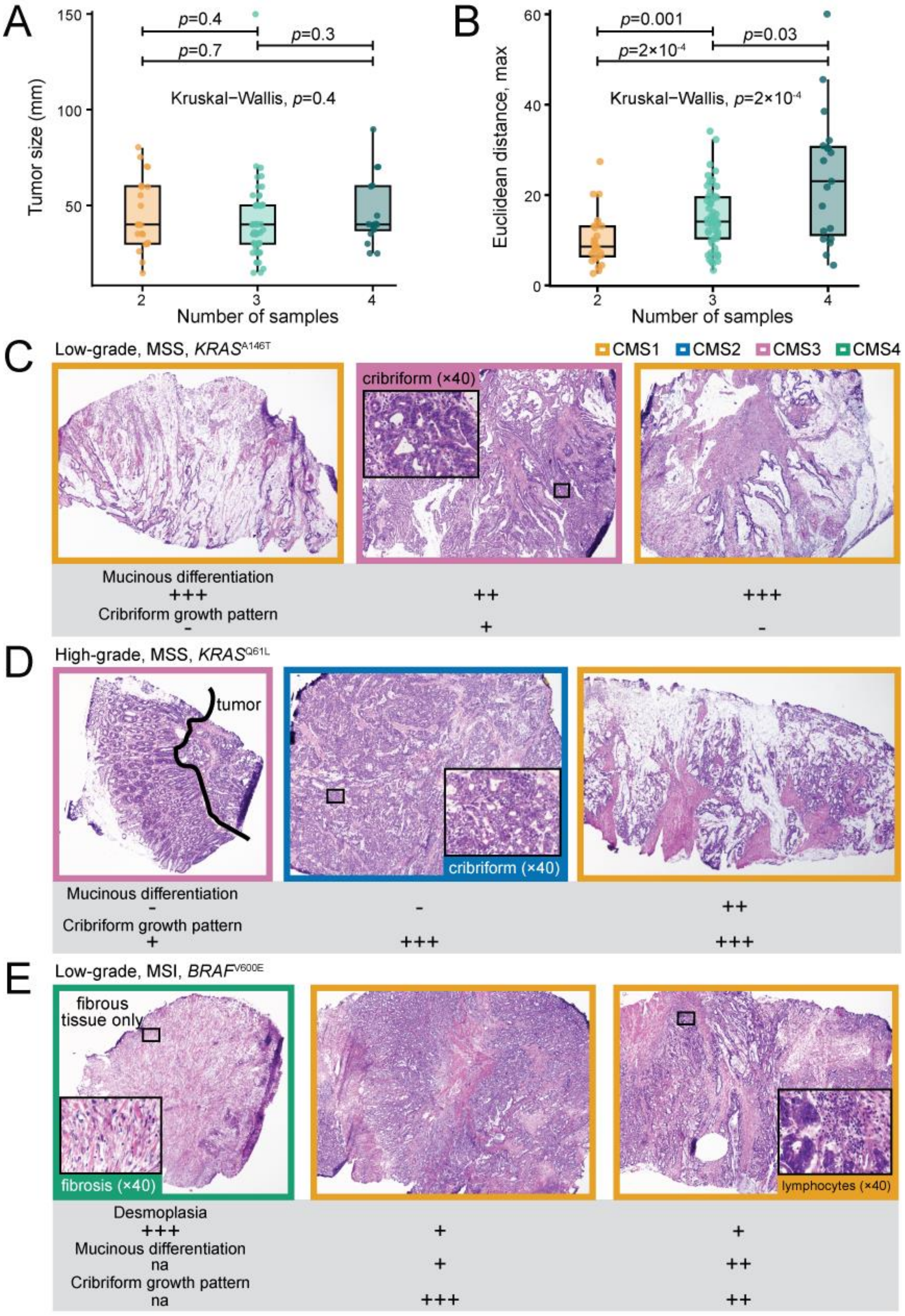

Supplementary Figure 2. Tumor size, sample number, general transcriptomic heterogeneity and histological cryosections of multiregional samples

Box plot of (A) tumor size (largest diameter in mm) and (B) general transcriptomic heterogeneity (estimated as the maximum Euclidean distance of principal components 1-3 between any pairs of

samples per tumor) according to the number of multiregional samples taken from each of $n = 98$ primary tumors. The center line of boxes represents the median, boxes represent the interquartile range, and whiskers represent 1.5× the interquartile range above the 75th percentile (maxima) or below the 25th percentile (minima). Pair-wise Wilcoxon test *p*-values between groups are shown in addition to the global Kruskal-Wallis test. Source data are provided as a Source Data file. C, D & E) Histological images (hematoxylin and eosin stains) of cryosections of three multiregional samples from each of three tumors with CMS heterogeneity (the cryosections were made of neighboring sections of the samples used for gene expression analyses). Insets show high-power images of selected areas (indicated in the smaller frames on low-power [×2] images). Morphological patterns previously associated with an image-based CMS classification were evaluated (Sirinukunwattana et al. Gut 2021;70:544-54), and patterns that differed among multiregional samples were indicated in grey boxes. The low-grade tumor in panel C showed mucinous differentiation, which was more extensive in the CMS1 samples than in the CMS3 sample. Only the CMS3 sample had cribriform growth patterns (shown in high-power inset). The high-grade tumor in panel D had mucinous differentiation only in the CMS1 sample, and prominent cribriform growth patterns in CMS2 in particular (shown in high-power inset). The CMS3 sample included normal mucosa, consistent with bulk transcriptomic classification as CMS3 rather than CMS2. The low-grade tumor in panel E had a CMS4 sample with fibrous tissue and prominent desmoplastic stromal reaction, as well as two samples with focal mucinous differentiation and classified as CMS1.

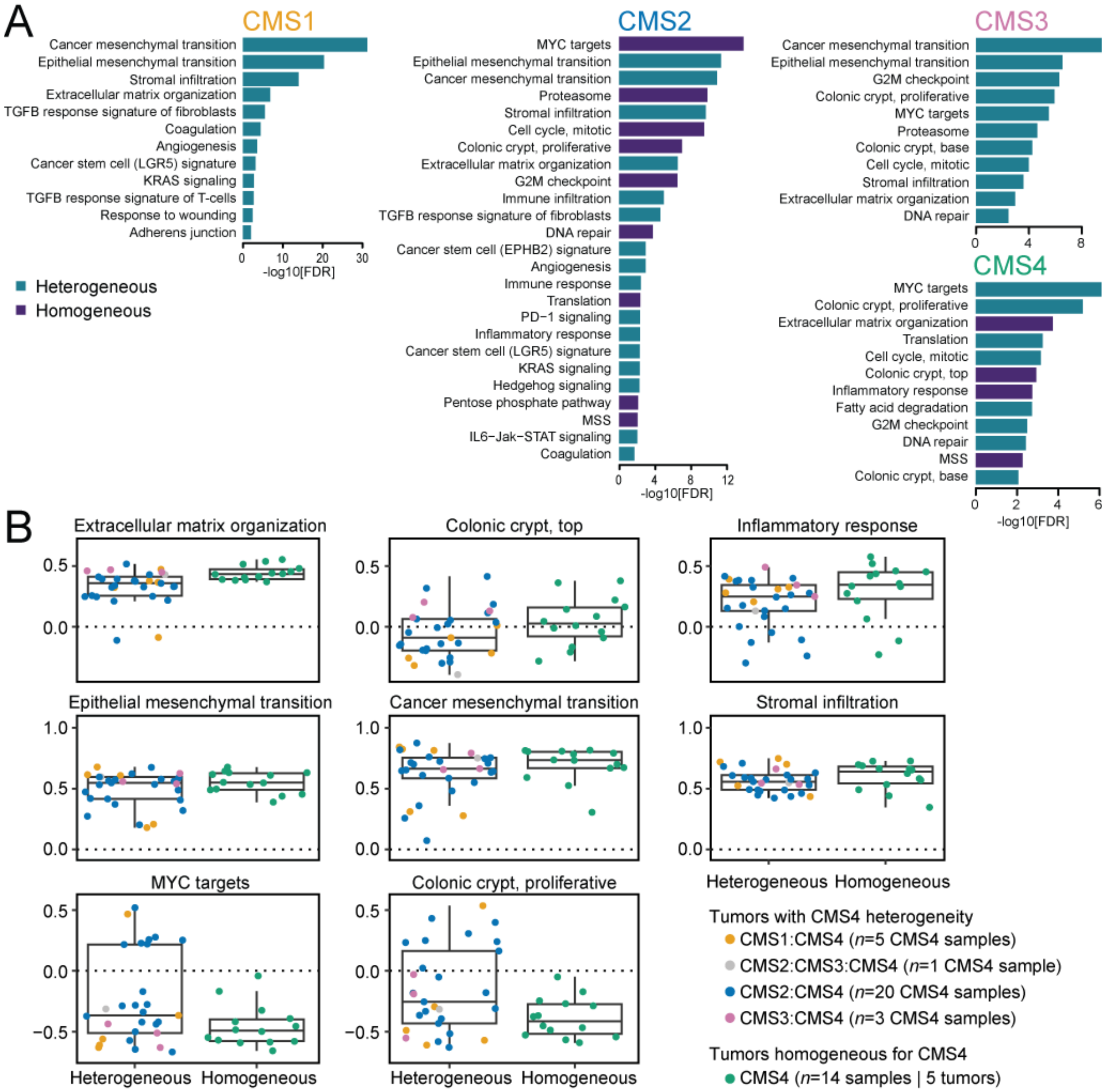

Supplementary Figure 3. Gene set enrichment analyses of heterogeneous versus homogeneous tumors for each CMS group in the multiregional sample set

A) Bar plots of FDR-adjusted *p*-values (log10-scale) for significant results (FDR < 0.05) from gene set enrichment analyses of tumors with homogeneous versus heterogeneous CMS classifications, performed within each of the CMS groups separately on the custom gene set collection (*n* = 54). Unclassified samples and samples from tumors with undetermined CMS-heterogeneity were excluded (total n = 266 samples, n_{CMS1} = 79, n_{CMS2} = 115, n_{CMS3} = 29 and n_{CMS4} = 43). Source data are provided in Supplementary Data 3. B) Box plots of single-sample enrichment scores (GSVA scores) of selected signatures in CMS4 samples from tumors with heterogeneous and homogeneous CMS4 classifications. All plotted samples are CMS4, but colored according to CMS combinations in heterogeneous tumors. The center line of boxes represents the median, boxes represent the interquartile range, and whiskers represent 1.5× the interquartile range above the 75th percentile (maxima) or below the 25th percentile (minima). Source data are provided as a Source Data file.

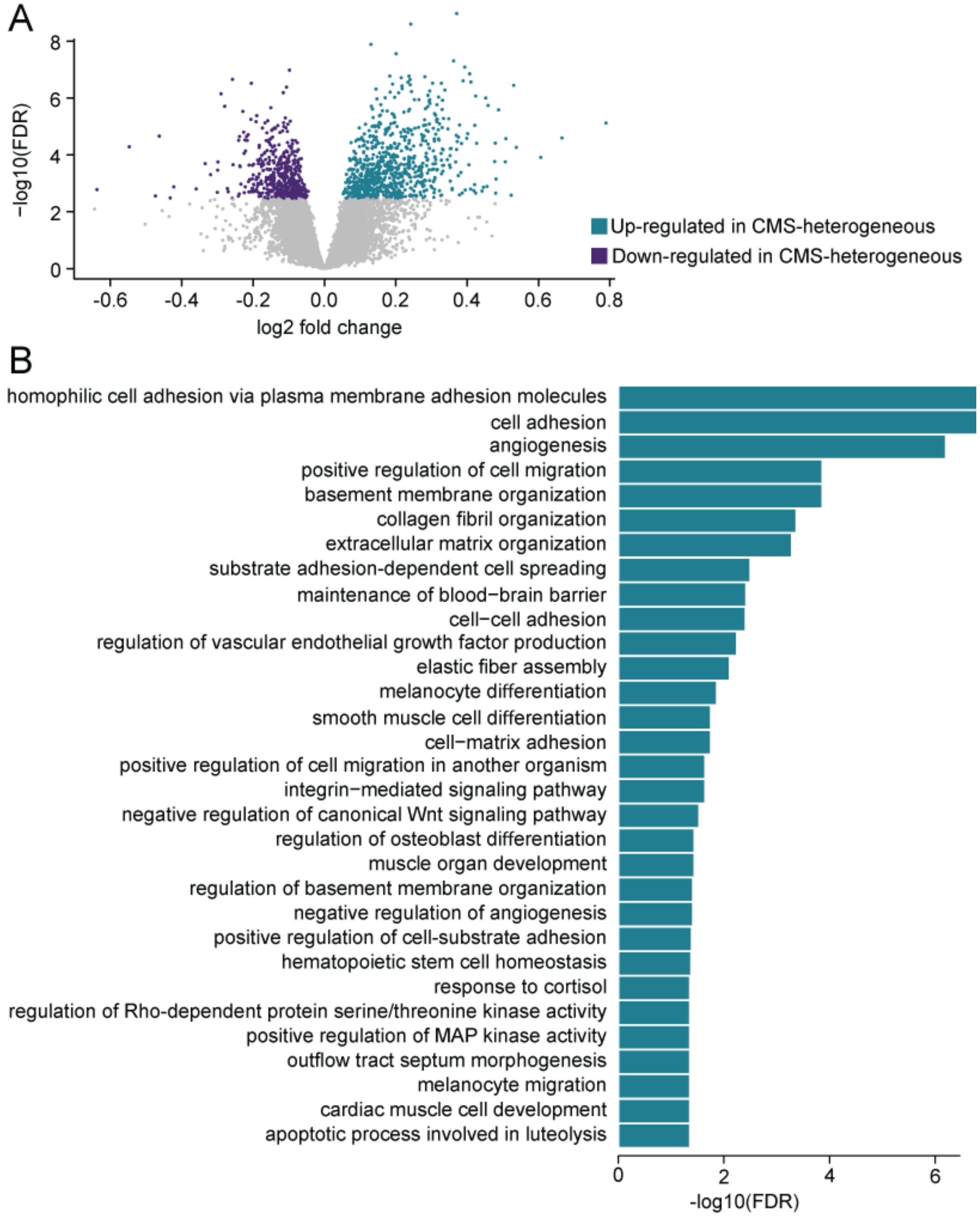

Supplementary Figure 4. Differentially expressed genes between tumors with heterogeneous versus homogeneous CMS classifications in the multiregional sample set

A) Volcano plot of differentially expressed genes (n_{up} = 805, n_{down} = 537, FDR < 0.05 from limma analysis) between tumors with heterogeneous and homogeneous intra-tumor classifications of multiregional samples (n = 270 samples; samples from tumors with undetermined CMS-heterogeneity were excluded). Source data are provided in Supplementary Data 4. B) Bar plot of significant (FDR < 0.05) enrichments of genes up-regulated in tumors with CMS heterogeneity (no significant enrichments for down-regulated genes) among ‘biological process’ terms from topGO analyses.

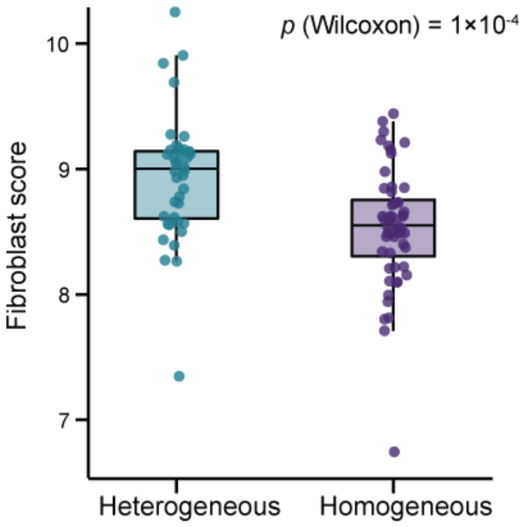

Supplementary Figure 5. Abundance of cancer associated fibroblasts in tumors with homogeneous and heterogeneous CMS classifications of multiregional samples

Box plot of cancer-associated fibroblast scores of heterogeneous and homogeneous tumors (total n = 98 tumors; the highest scoring sample was plotted for each tumor). The center line of boxes represents the median, boxes represent the interquartile range, and whiskers represent 1.5× the interquartile range above the 75th percentile (maxima) or below the 25th percentile (minima). Source data are provided as a Source Data file.

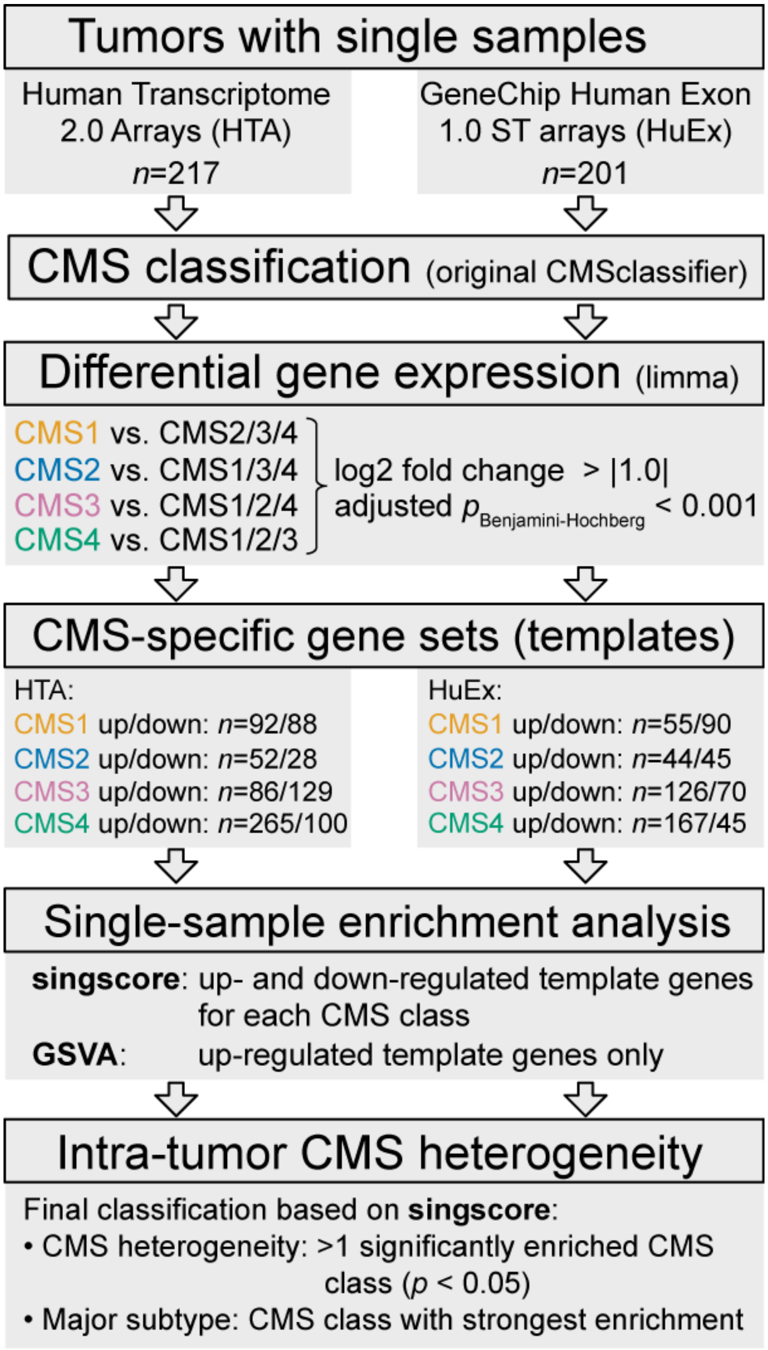

Supplementary Figure 6. Overview of computational approach for estimation of intra-tumor CMS heterogeneity in primary CRCs with a single sample

Single samples from 418 primary tumors were classified using the original random forest CMSclassifier (posterior probability threshold 0.4). HTA and HuEx samples were analyzed separately. Differential gene expression analysis of each CMS class was used to identify CMS-specific gene lists. Gene set enrichment analysis of each gene list in each sample was performed with two algorithms and singscore was used for further analyses. Samples with significant enrichments for more than one CMS class (singscore $p < 0.05$) were considered to have intra-tumor CMS heterogeneity.

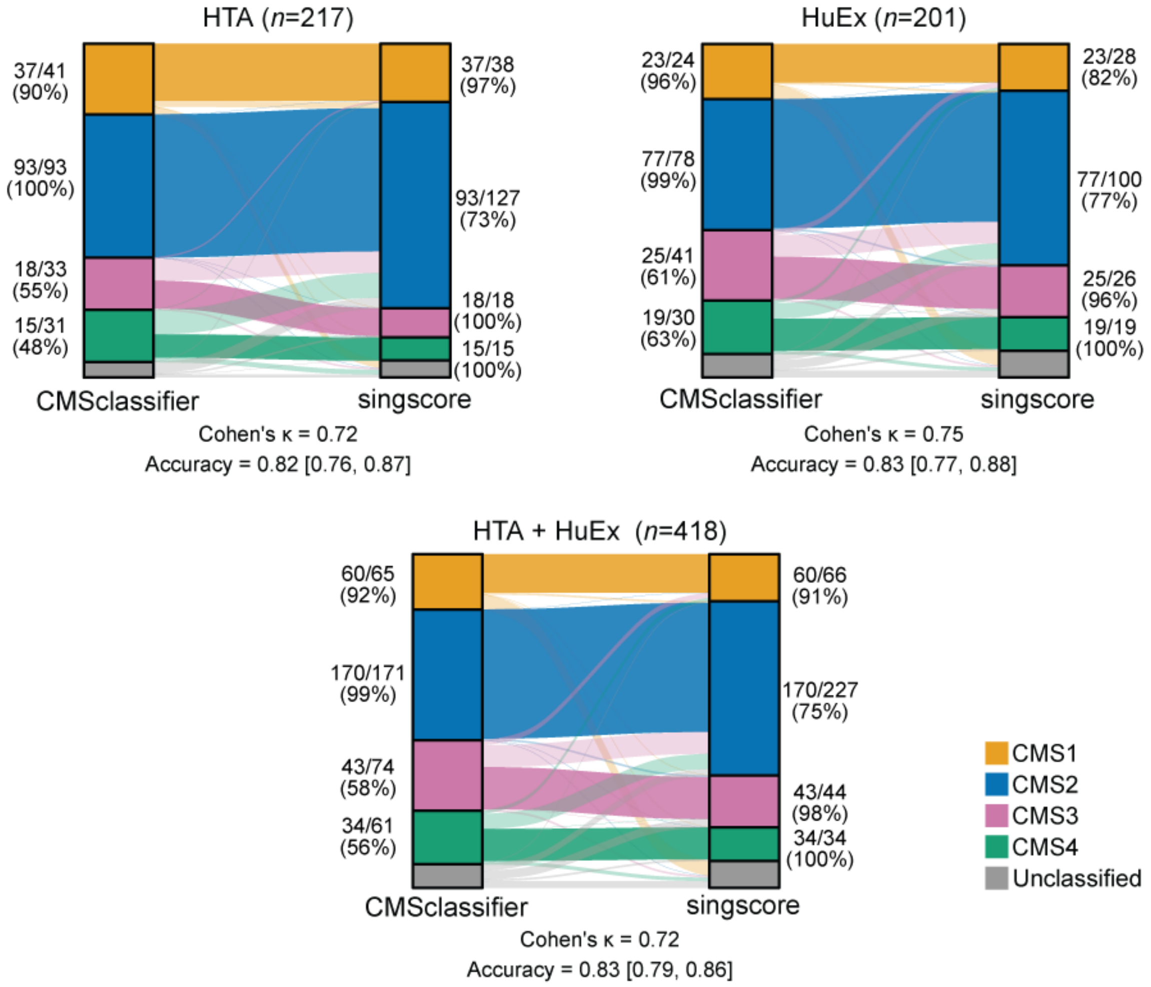

Supplementary Figure 7. Concordance of CMS classifications according to the original classifier and singscore enrichments in the single-sample primary tumor set

Alluvial plots are shown for tumors analyzed on HTA arrays (left; n = 217), HuEx arrays (right; n = 201) and both combined (bottom; n = 418). Cohen's κ and accuracy with 95% confidence intervals are indicated for all sample sets. Colors are according to the original CMSclassifier using a posterior probability threshold of 0.4. Source data are provided as a Source Data file.

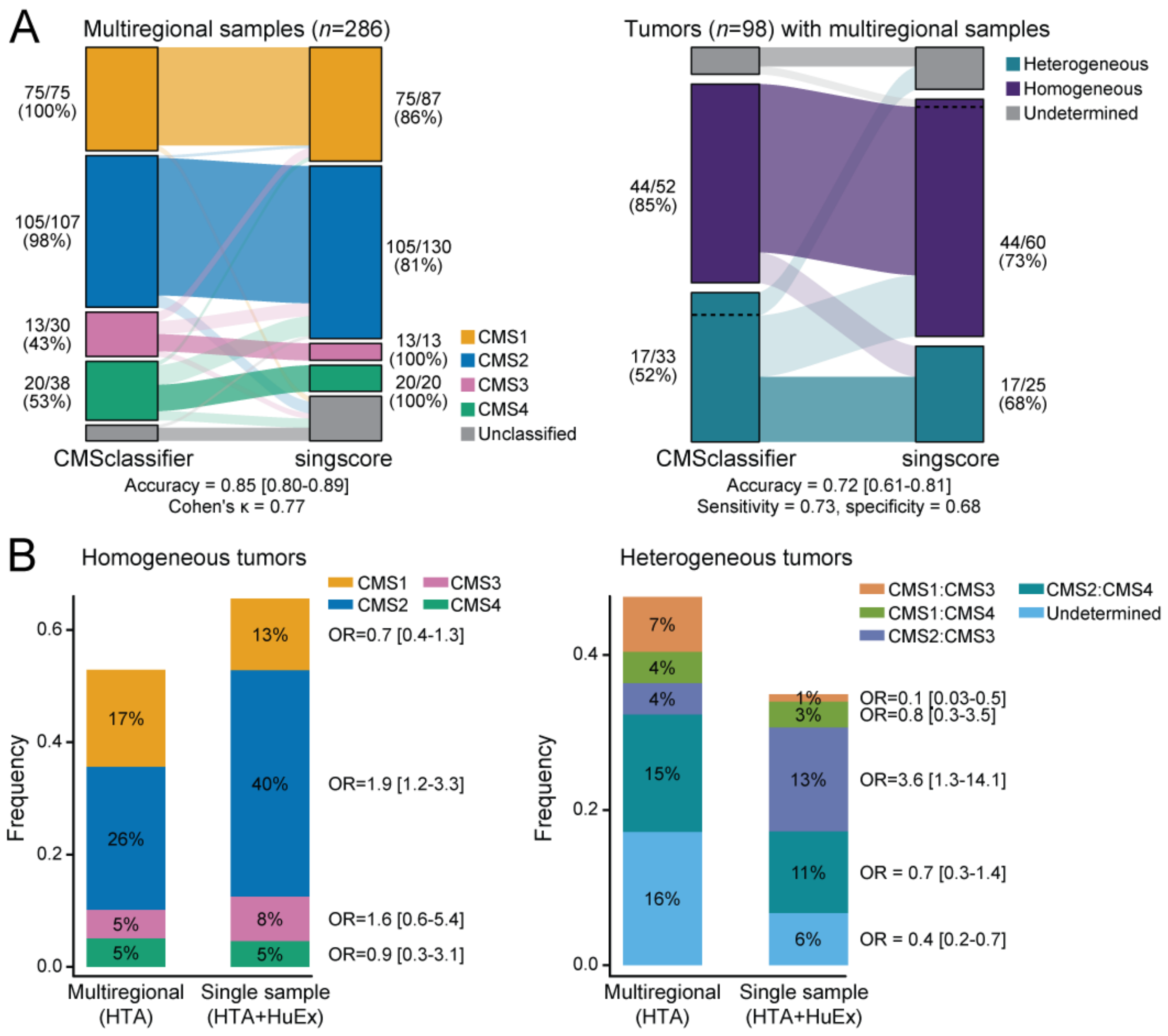

Supplementary Figure 8. Evaluation of computational approach for classification of intra-tumor CMS heterogeneity of single samples

A) Alluvial plots for CMS classification of multiregional samples (left) and scoring of intra-tumor CMS heterogeneity among multiregional samples (right) using the original CMSclassifier versus singscore enrichments. The singscore enrichments were based on CMS template gene sets derived from the independent single-sample tumor series. Tumors were considered heterogeneous according to singscore if at least one sample was heterogeneous, and tumors with different (homogeneous) CMS classes in different samples were not considered heterogeneous (to disregard the spatial resolution and evaluate the computational intra-sample analysis only). The proportion of overlapping samples was estimated among tumors with determined heterogeneity status only. Source data are provided as a Source Data file. B) CMS frequencies in homogeneous tumors and CMS combinations in heterogeneous tumors plotted separately for the multiregional sample set (*n* = 98 tumors; observed CMS heterogeneity) and single-sample primary tumors (*n* = 418; computational estimation of CMS heterogeneity). Odds ratios (OR) with 95% confidence intervals were calculated by Fisher's exact test. Source data are provided as a Source Data file.

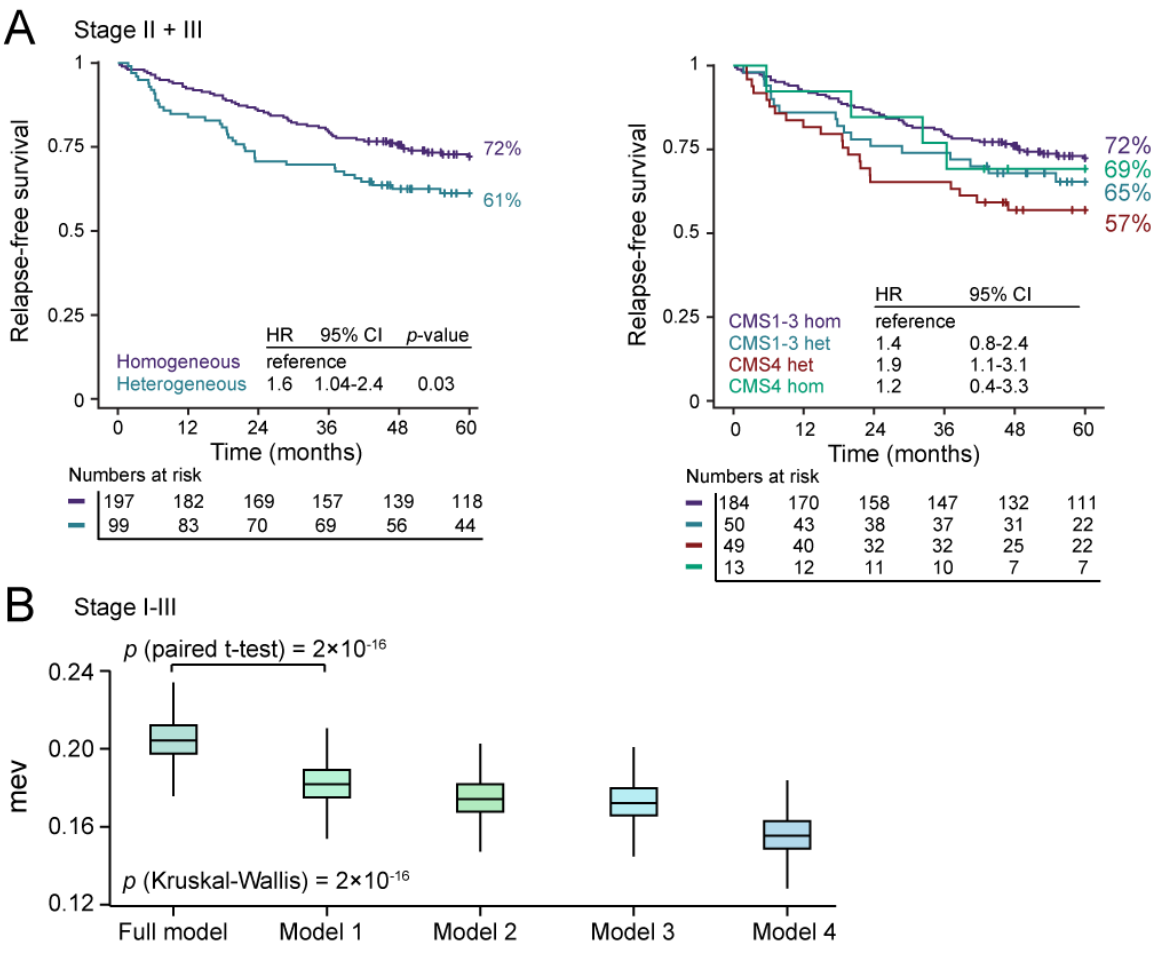

Model	Model variable(s)	Mean mev [95% CI]
Full model:	Clin + genomic + CTL + CAF + CMS-het	0.21 [0.19-0.24]
Model 1:	Clin + genomic + CTL + CAF	0.18 [0.16-0.21]
Model 2:	Clin + genomic + CTL	0.17 [0.15-0.19]
Model 3:	Clin + genomic	0.17 [0.15-0.19]
Model 4:	Clin	0.15 [0.12-0.17]

Supplementary Figure 9. Five-year relapse-free survival according to intra-tumor CMS heterogeneity

A) Kaplan-Meier curves of intra-tumor CMS heterogeneity (left) and CMS heterogeneity stratified by CMS4 classification (right) among patients treated by complete resection of stages II and III CRC (*n* = 296). Patients with synchronous tumors, pre-surgical radiation treatment and undetermined CMS heterogeneity were excluded from analyses. Hazard ratios (HR) and 95% confidence intervals (CI) are from Cox proportional hazards analyses and *p*-values from Wald tests. B) Box plot of bootstrapped measures (*n* = 5,000 iterations) of explained variation (mev) for Cox proportional hazards models among patients treated by complete resection of stage I-III CRC (*n* = 387) and including the following variables: Clin, clinicopathological parameters (age, sex, TN stage, localization, adjuvant chemotherapy); genomic (MSI/KRAS/BRAF-status); CTL, cytotoxic lymphocyte score; CAF, cancer-associated fibroblast score; CMS-het, CMS heterogeneity. The center line of boxes represents the median, boxes represent the interquartile range, and whiskers represent 1.5× the interquartile range above the 75th percentile (maxima) or below the 25th percentile (minima).

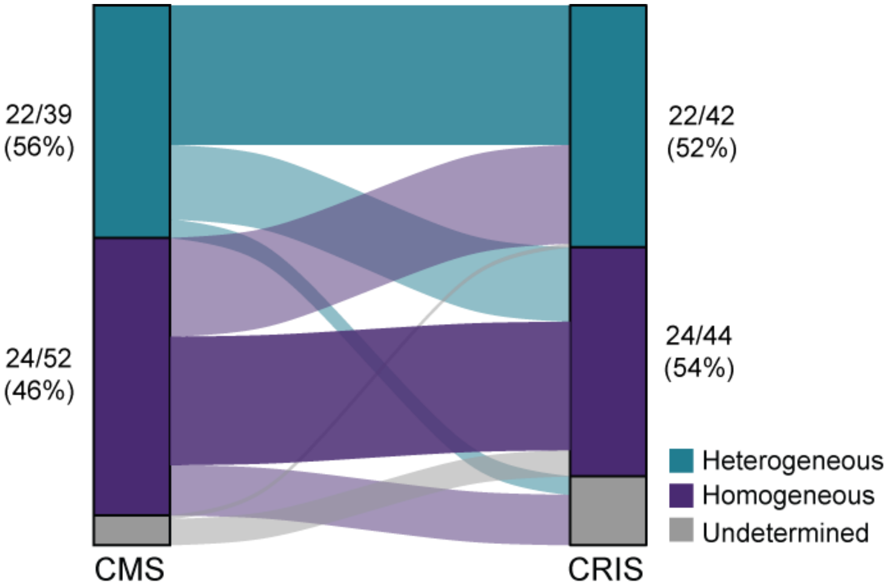

Supplementary Figure 10. Limited concordance in intra-tumor heterogeneity of CMS and CRIS classifications

Alluvial plot illustrates the limited overlap of tumors in the multiregional sample set (*n* = 98) classified as heterogeneous or homogeneous in the CMS and CRIS frameworks. The overlap is indicated relative to the total number of tumors per group. Source data are provided as a Source Data file.

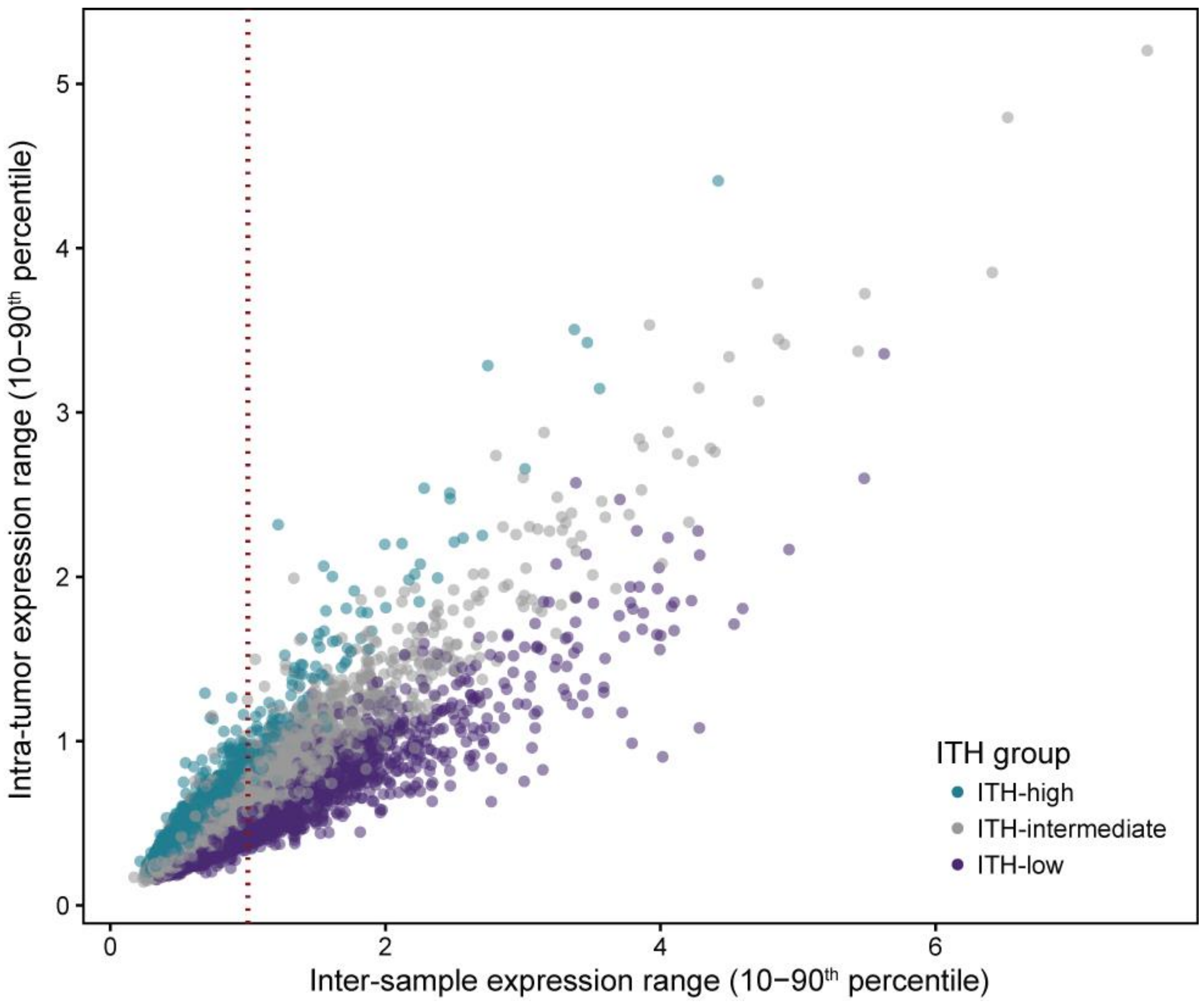

Supplementary Figure 11. Intra-tumor versus inter-sample gene expression variation in the multiregional sample set

Scatterplot of the intra-tumor versus inter-sample expression range (10-90th percentile ranges) of all protein-coding genes (*n* = 18,823) in the multiregional sample set (*n* = 286 samples and 98 tumors). Genes are colored according to three categories of intra-tumor heterogeneity, based on the custom thresholds for the ITH-score. The vertical red dashed line indicates the gene filtering threshold based on inter-sample variation (10-90th percentile range of 1). Source data are provided as a Source Data file.

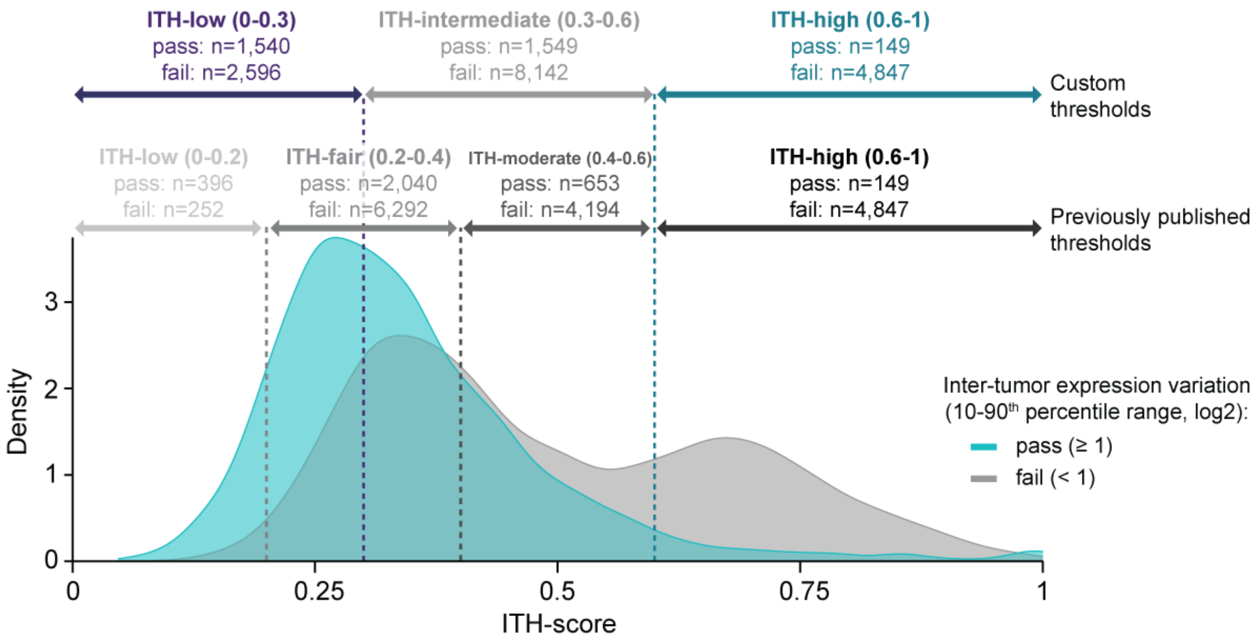

Supplementary Figure 12. Distribution of genes according to ITH-score

Density plot of ITH-scores of all protein-coding genes (*n* = 18,823) grouped according to the inter-sample expression variation filter in the multiregional sample set (10-90th percentile range above or below 1). Categorization of genes according to the ITH-score using custom and previously published thresholds (Gyanchandani et al. Clin Cancer Res 2016;22:5362-9) are indicated by dashed lines. The number of genes that passed or failed the inter-sample expression variation threshold is indicated for all groups. Source data are provided as a Source Data file.

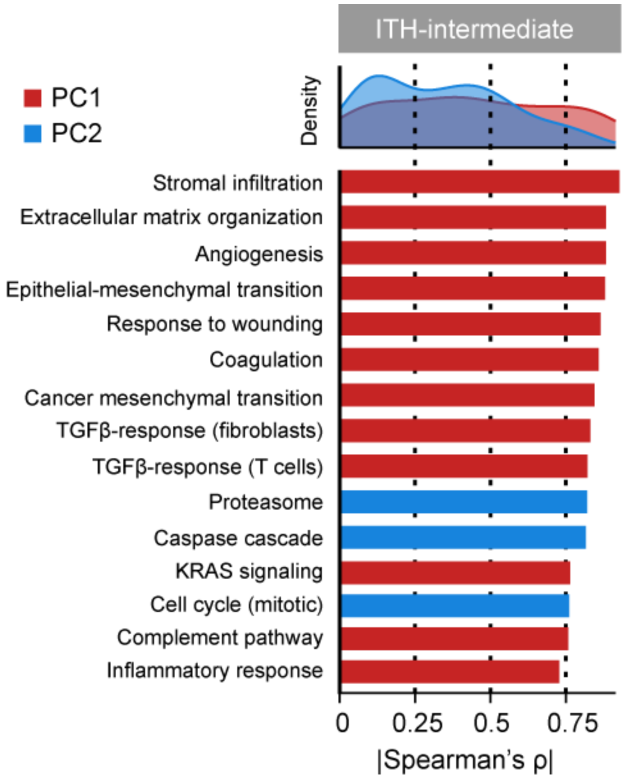

Supplementary Figure 13. Gene set correlations of ITH-intermediate genes

Density plots (upper: all gene sets, n = 54) and bar plots (lower: 15 top-ranked gene sets) of Spearman's correlation coefficients (absolute values) between principal components (PC1 or PC2) of ITH-intermediate genes and single-sample enrichment scores of significantly correlated gene sets ($p < 0.05$) in the multiregional sample set (n = 286 primary tumor samples). Source data are provided as a Source Data file.

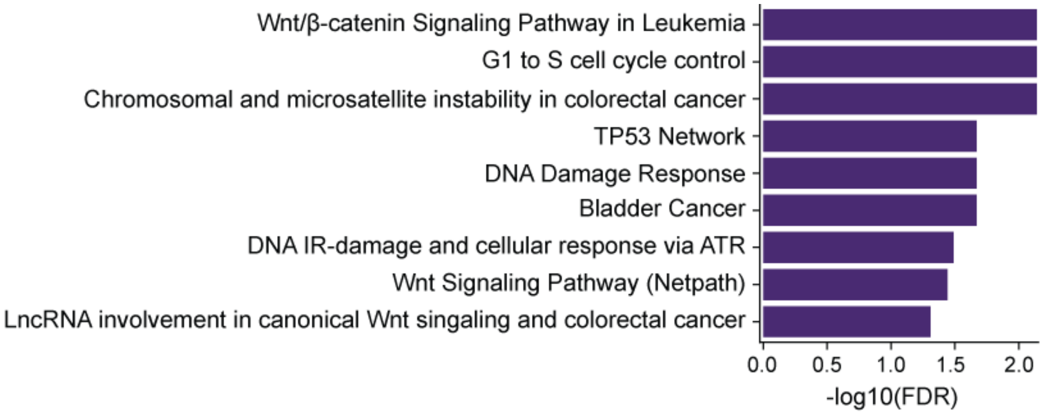

Supplementary Figure 14. Gene set overrepresentation analysis of ITH-low cancer-critical genes

Pathways from the Wikipathway cancer database significantly over-represented (FDR < 0.05) among ITH-low cancer-critical genes (genes included in the Cancer Gene Census) using the WEB-based Gene SeT AnaLysis Toolkit. No significant over-representation was found for ITH-high or ITH-intermediate genes. Source data are provided as a Source Data file.

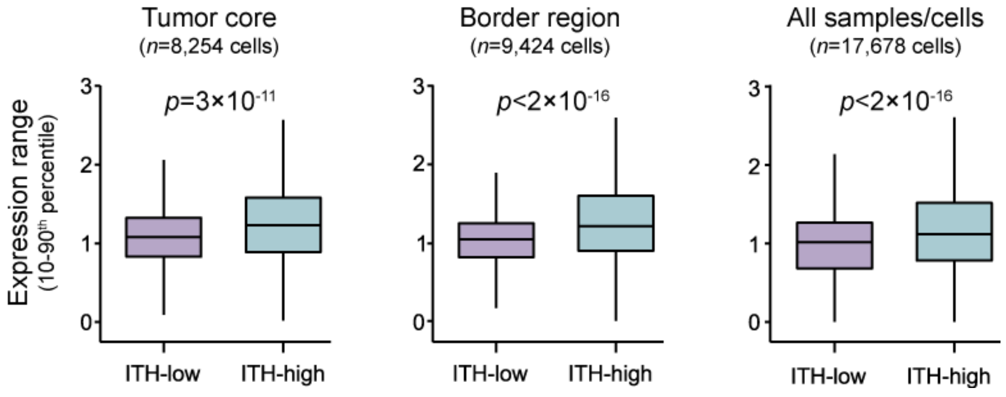

Supplementary Figure 15. Validation of ITH-high and ITH-low gene categories in single-cell RNA sequencing data

Single-cell RNA sequencing data of 12 paired samples from 6 primary CRCs (representing the tumor core and border regions) were downloaded from GSE144735. Box plots show the 10-90th percentile expression range of ITH-high and ITH-low genes in cells from the tumor core, border regions and both (*p*-values from Welch's t-tests). The center line of boxes represents the median, boxes represent the interquartile range, and whiskers represent 1.5× the interquartile range above the 75th percentile (maxima) or below the 25th percentile (minima).

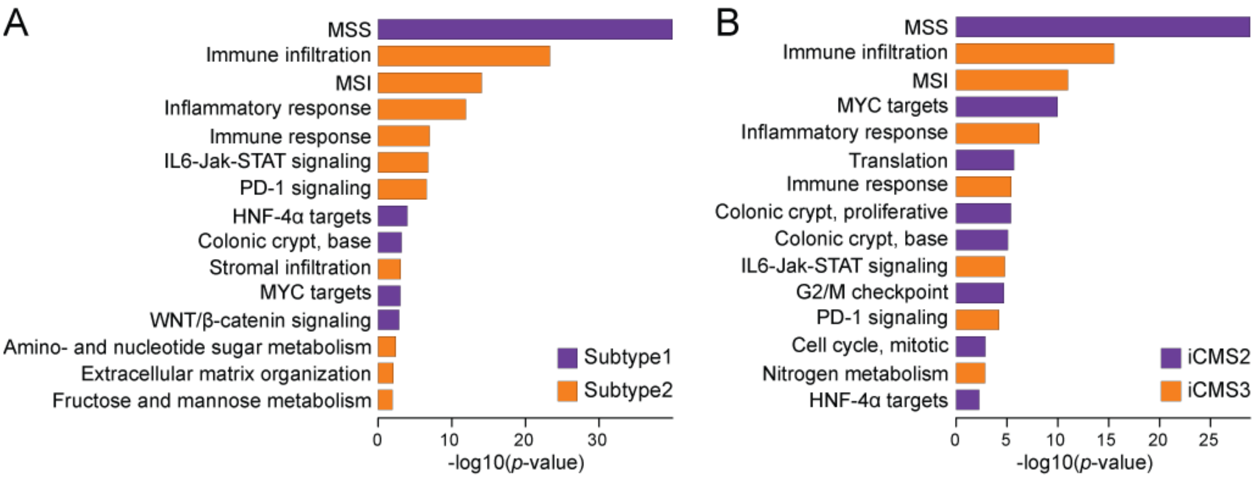

Supplementary Figure 16. Gene set enrichment analyses of subtypes in the ITH-low k2 and iCMS frameworks

Bar plots of the 15 top-ranked gene sets from a custom gene set collection (n = 54) ranked based on statistical significance from gene set enrichment analyses comparing subtypes of primary CRCs classified according to (A) k2 from NMF of ITH-low genes (n = 704 classified samples) and (B) iCMS (n = 643 classified samples). Source data are provided as a Source Data file.

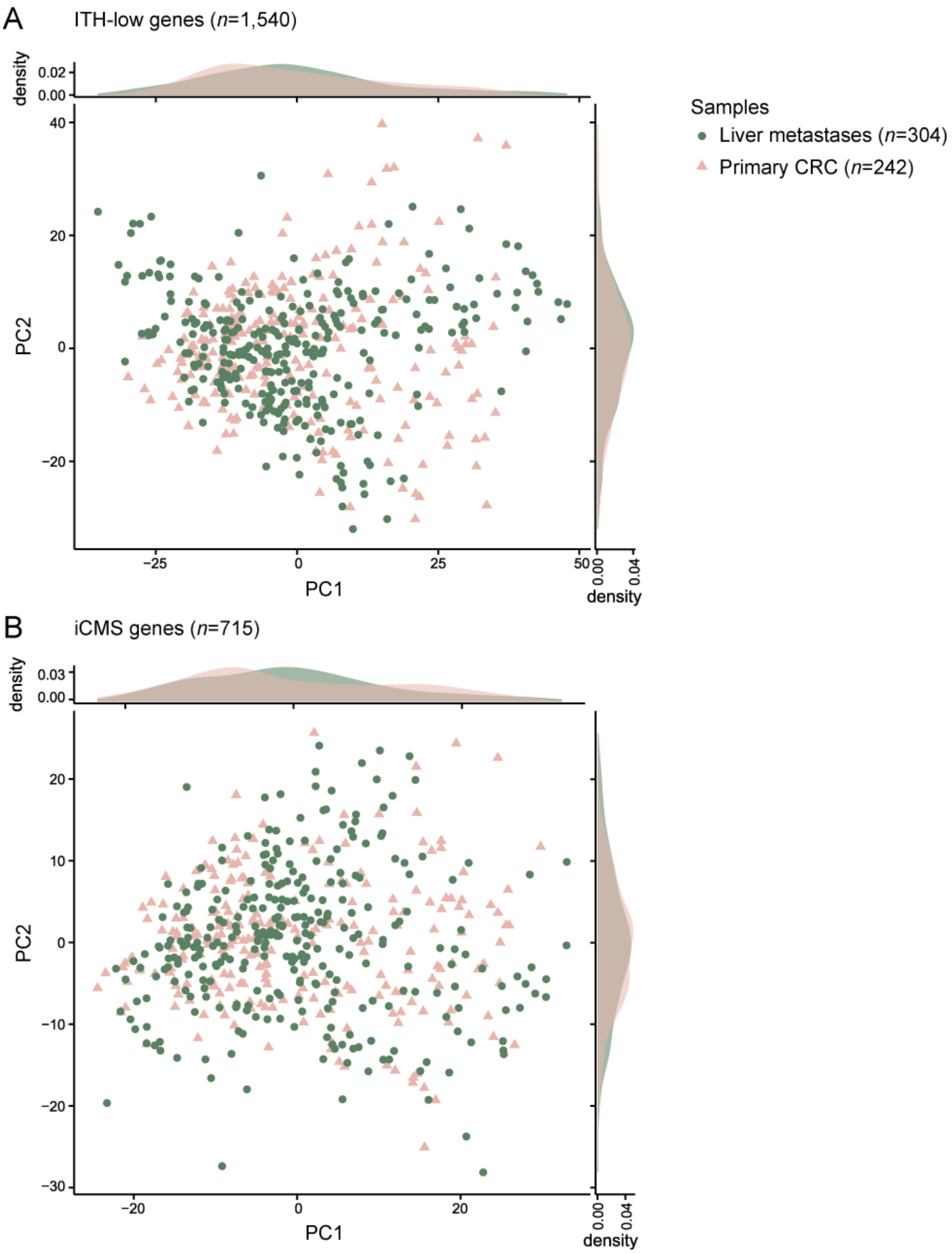

Supplementary Figure 17. Principal components analysis of primary CRC and liver metastasis samples

PCA plot based on the expression level of (A) ITH-low genes (from multiregional primary tumor samples) and (B) iCMS template genes. Density plots show the distribution of PC1 and PC2 for primary CRC (pink) and liver metastases (green) samples separately. Source data are provided as a Source Data file.

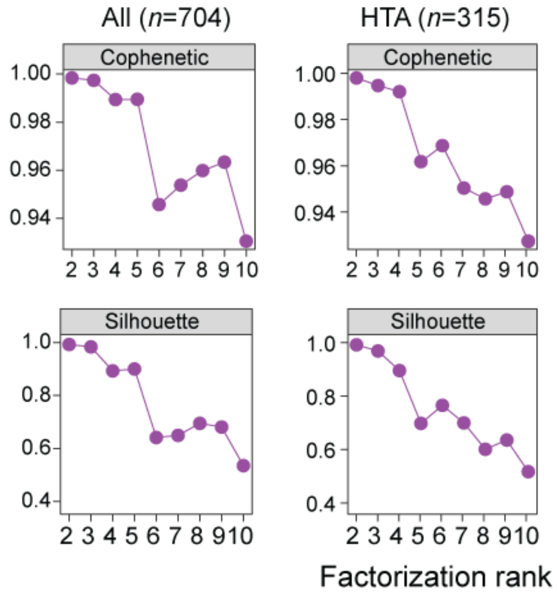

Supplementary Figure 18. Cophenetic and silhouette scores from NMF clustering of different sample sets with a predefined interval of factorization ranks

Three different sample sets were analyzed by NMF based on ITH-low genes, including all primary tumor samples (left), one randomly selected sample from each primary tumor analyzed on HTA only (middle), and one randomly selected sample from each primary MSS tumor (analyzed on HTA or HuEx; right).

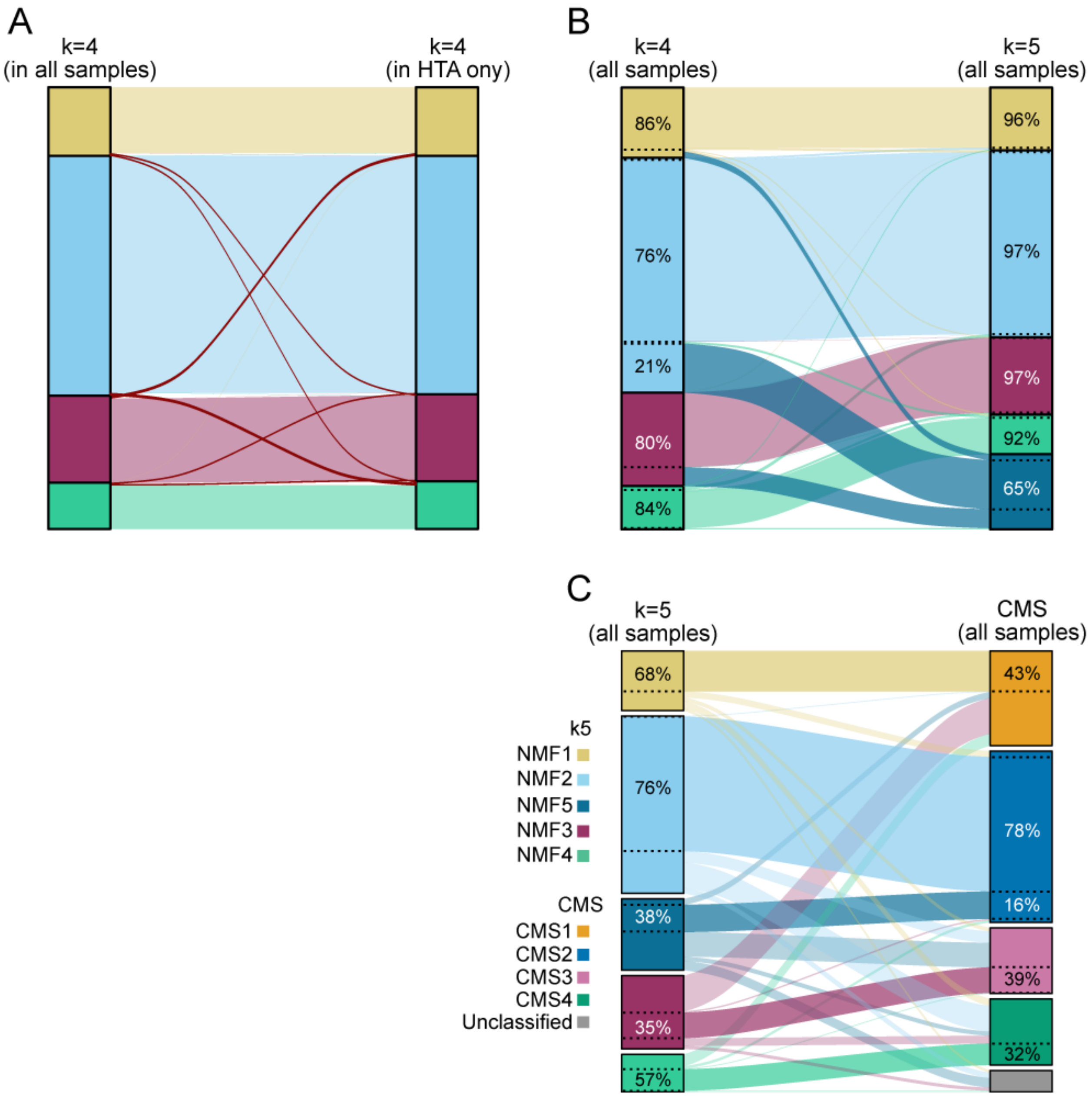

Supplementary Figure 19. Alluvial plots of concordance in NMF classification between sample sets

A) Sample clustering at *k*=4 in the complete primary tumor sample set (left) and of single, randomly selected samples from each tumor analyzed on HTA (right). Only overlapping samples between data sets are plotted (*n* = 315 tumors) and discordant classifications are indicated in dark red. Source data are provided as a Source Data file. B) Sample clustering at *k*=4 (left) and *k*=5 (right) in the complete primary tumor sample set (*n* = 704 samples). The largest cluster from *k*=4 (light blue) was primarily subdivided into two clusters (light blue and dark blue) at *k*=5. Source data are provided as a Source Data file. C) Same as panel B, but the k5 clusters based on ITH-low genes are compared with the original CMS classification. Source data are provided as a Source Data file.

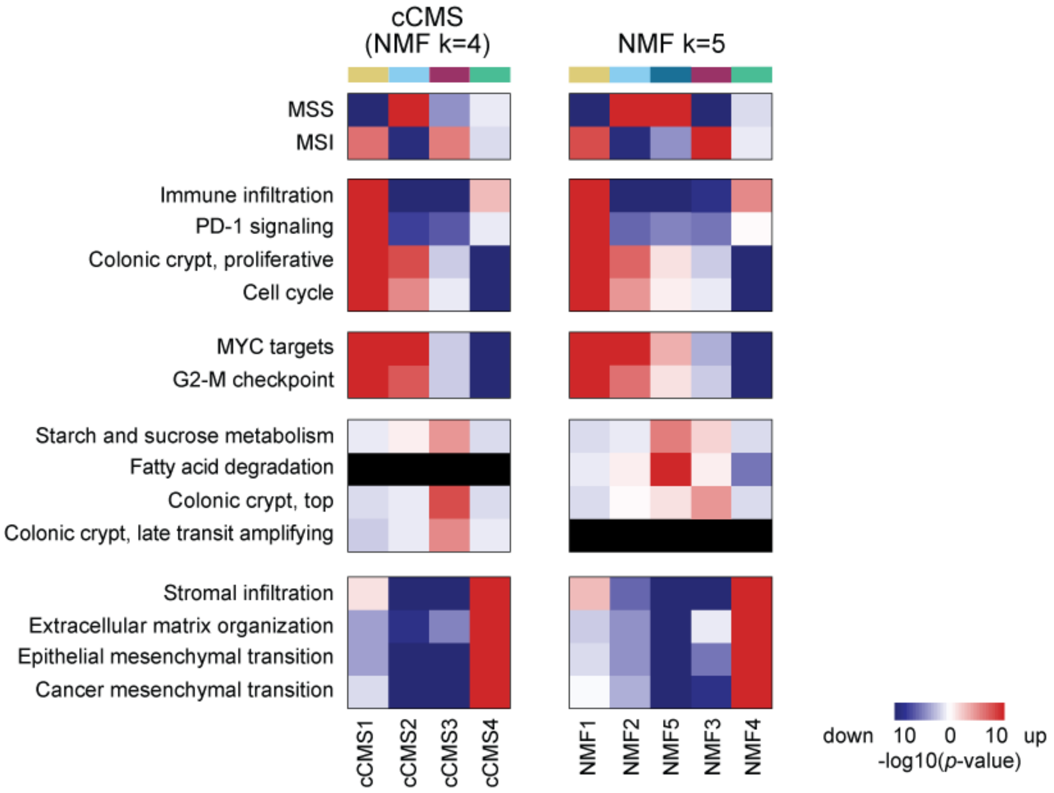

Supplementary Figure 20. Gene set enrichment analyses of subtypes based on ITH-low genes

Heat map of *p*-values (log10-scale) of significant enrichments (red: positive enrichments; blue: negative enrichments) from the custom gene set collection according to NMF classification at k=4 (cCMS) and k=5 clusters across all primary tumor samples (n = 704 samples from 516 tumors). The two clusters at k=5 (NMF2 and NMF5) that were derived primarily from the blue cluster at k=4 (Supplementary Figure 19B) showed highly similar gene expression enrichments, and were plotted adjacently. Source data are provided as a Source Data file.

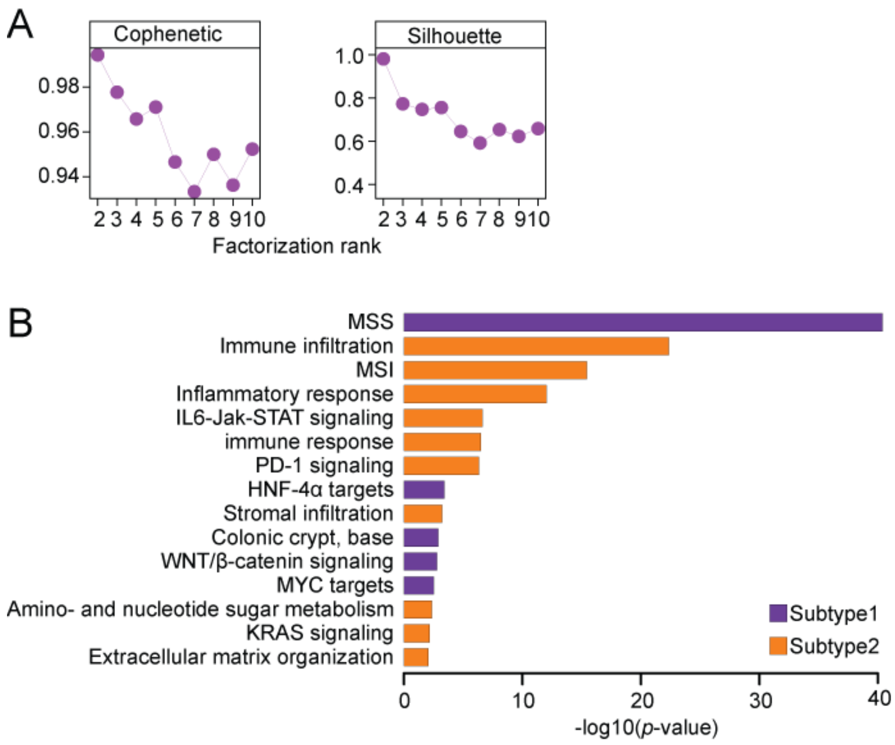

Supplementary Figure 21. NMF classification based on ITH-low genes defined by previously published threshold

NMF was also tested on all primary tumor samples (n = 704) based on ITH-low genes defined as ITH-score ≤ 0.2 (n = 396 genes). A) Both the cophenetic coefficient (left) and silhouette width (right) indicated k=2 as the optimal number of sample clusters. B) Bar plot of top 15 gene sets ranked based on statistical significance from gene set enrichment analysis of the custom gene set collection comparing the two sample clusters from NMF at k=2. MSS-like and MSI-like expression characteristics were the main discriminatory features between the sample clusters. Source data are provided as a Source Data file.

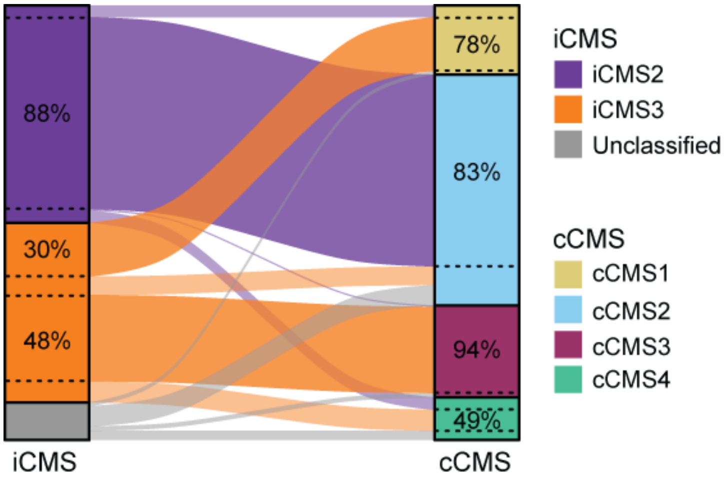

Supplementary Figure 22. Classification concordance between iCMS and cCMS

Alluvial diagram illustrating concordance of iCMS and cCMS classifications in the complete primary tumor series (*n* = 704 samples). The sample overlap is indicated relative to the total sample number per subtype. 9% of samples were unclassified for iCMS. Source data are provided as a Source Data file.

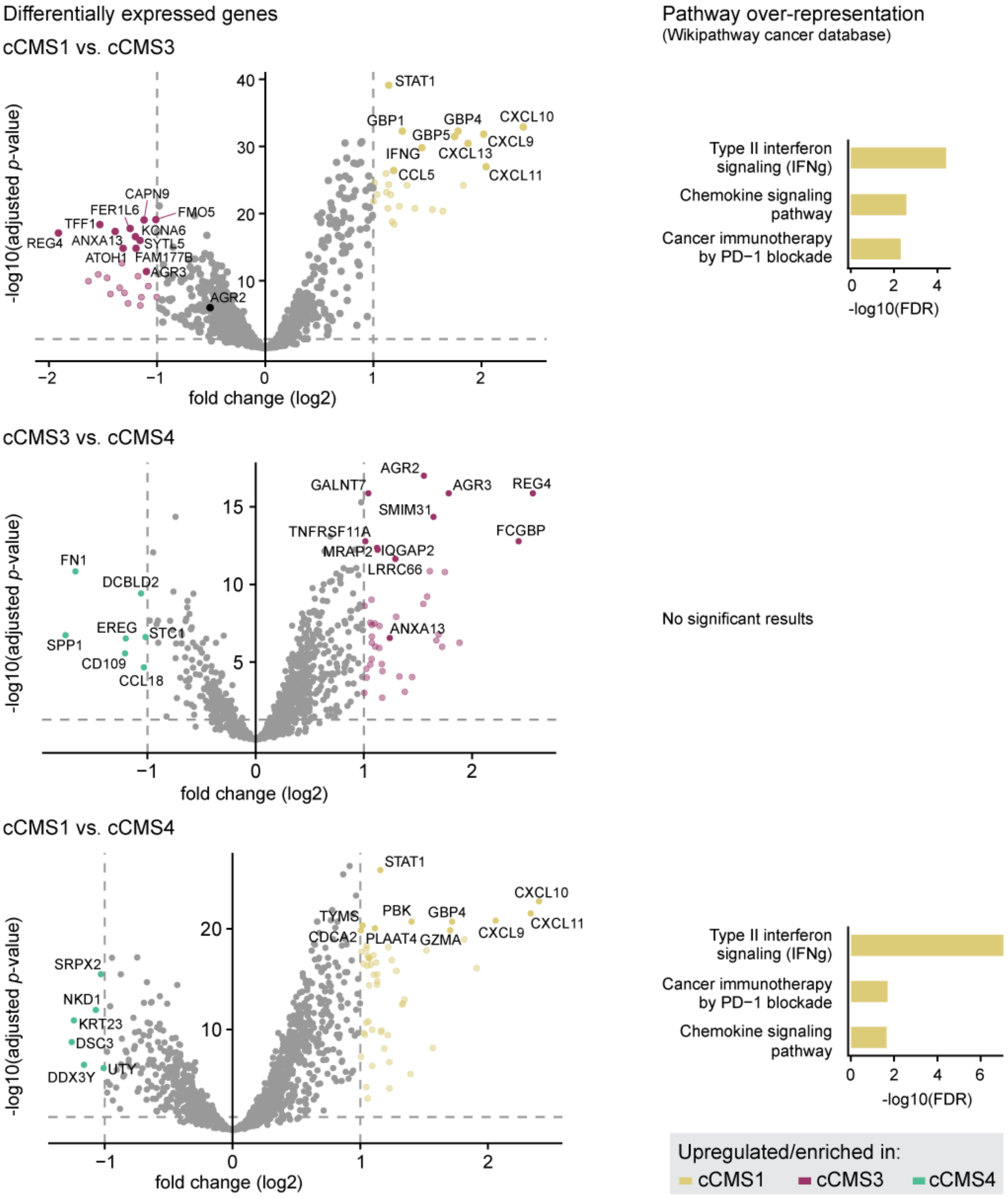

Supplementary Figure 23. Differential expression of ITH-low genes among sample clusters split from the k2 cluster corresponding to iCMS3

The k2 sample cluster corresponding to iCMS3 were split into the k4 clusters cCMS1 (*n* = 96 samples), cCMS3 (*n* = 139) and cCMS4 (*n* = 37; Figure 5b). Volcano plots (left) illustrate results from differential

expression analysis of ITH-low genes (n = 1,540) between the sample groups as indicated. Genes with significant differential expression (adjusted p-value < 0.05 and log2 fold change>|1| from limma analysis, indicated by dashed lines) are highlighted by corresponding colors. The top 10 (by adjusted p-value) and selected genes are annotated. Bar plots (right) show all significant pathway enrichments (FDR < 0.05) of the differentially expressed genes in the Wikipathway cancer database, evaluated by the WEB-based Gene SeT AnaLysis Toolkit. Source data are provided as a Source Data file.

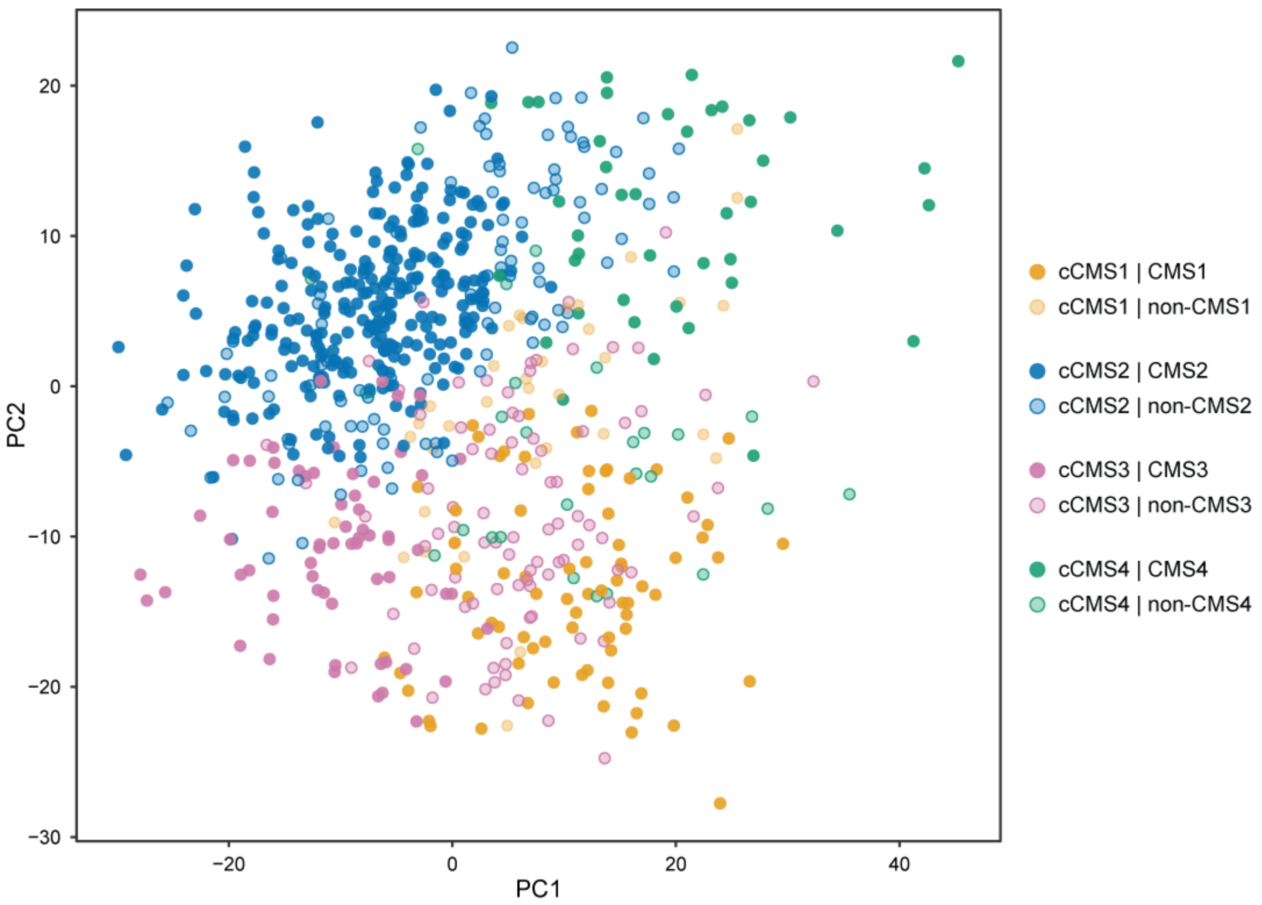

Supplementary Figure 24. PCA plot comparing the CMS and cCMS classification frameworks

PCA was performed of the *n* = 1,000 genes with highest expression variance across all primary tumor samples (*n* = 704). Samples are plotted and colored according to cCMS and CMS classifications. Samples with discordant classifications between the two frameworks are colored with lower opacity (for example, cCMS1 samples that are not CMS1 are colored in transparent yellow). Source data are provided as a Source Data file.

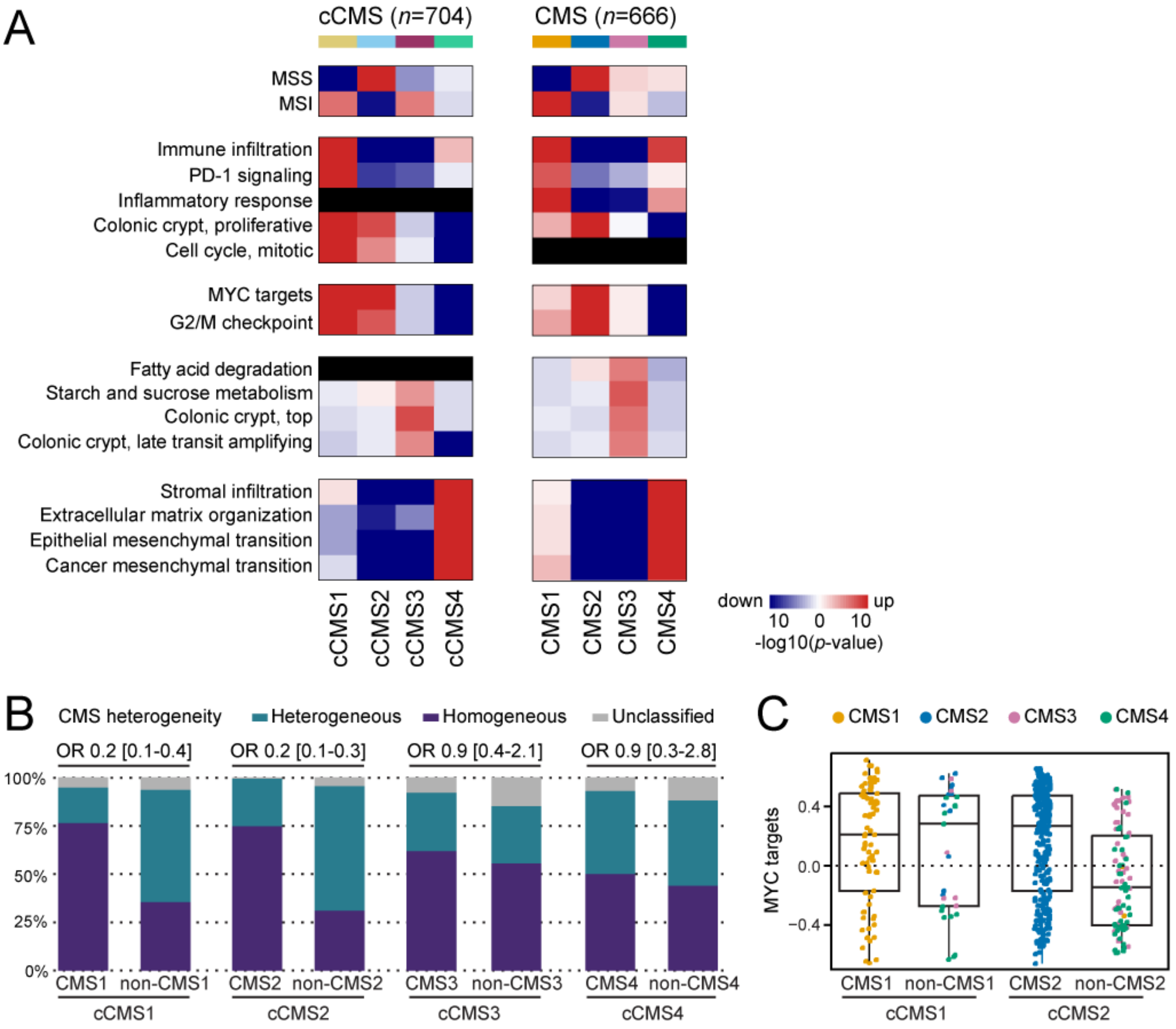

Supplementary Figure 25. Gene set enrichment analyses according to cCMS and CMS classifications

A) Heat map of *p*-values (log10-scale) of significant enrichments (red: positive enrichments; blue: negative enrichments) from gene set enrichment analyses of the custom gene set collection (*n* = 54 gene sets) according to NMF classification based on ITH-low genes at *k*=4 (cCMS; *n* = 704 classified primary tumor samples) and the original CMS classification (*n* = 666 classified primary tumor samples). Source data are provided as a Source Data file. B) Proportion of samples from CMS heterogeneous tumors in each cCMS class, grouped according to classification concordance with the original CMS framework. cCMS1 and cCMS2 samples that were not of the corresponding original CMS class were more frequently from tumors with CMS heterogeneity. Samples with unknown CMS classification were not included in the plot. Odds ratios (ORs) and 95% confidence intervals are from Fisher's exact test of heterogeneous versus homogeneous tumors in each cCMS class. Source data are provided as a Source Data file. C) The MYC target signature was not enriched in cCMS2 samples compared to cCMS1, and had low scores in cCMS2 samples that were not of the corresponding CMS class. The center line of boxes represents the median, boxes represent the interquartile range, and whiskers represent 1.5× the interquartile range above the 75th percentile (maxima) or below the 25th percentile (minima). Source data are provided as a Source Data file.

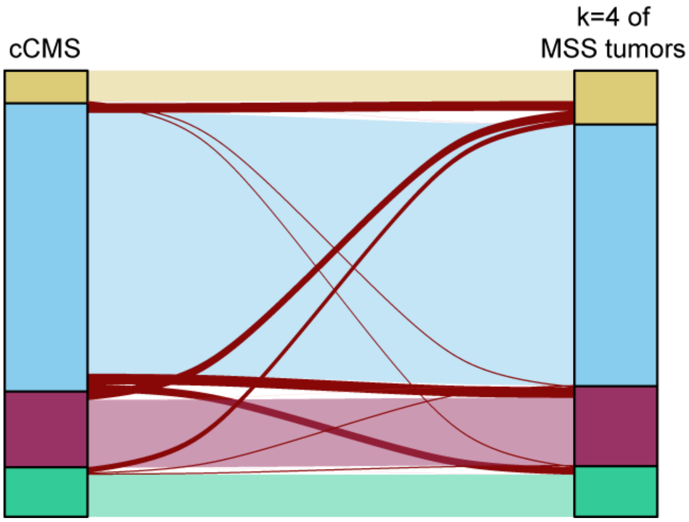

Supplementary Figure 26. Classification concordance from subtype discovery of all primary CRC samples versus MSS samples only

Alluvial plots of the classification concordance of primary MSS CRC samples (n = 424) based on cCMS (using all primary tumor samples for subtype discovery, including both MSI and MSS) and a repeated subtype discovery of MSS samples only. Discordant classifications are marked in dark red. Subtype discovery was performed with NMF at k=4 on ITH-low genes in both sample sets. Source data are provided as a Source Data file.

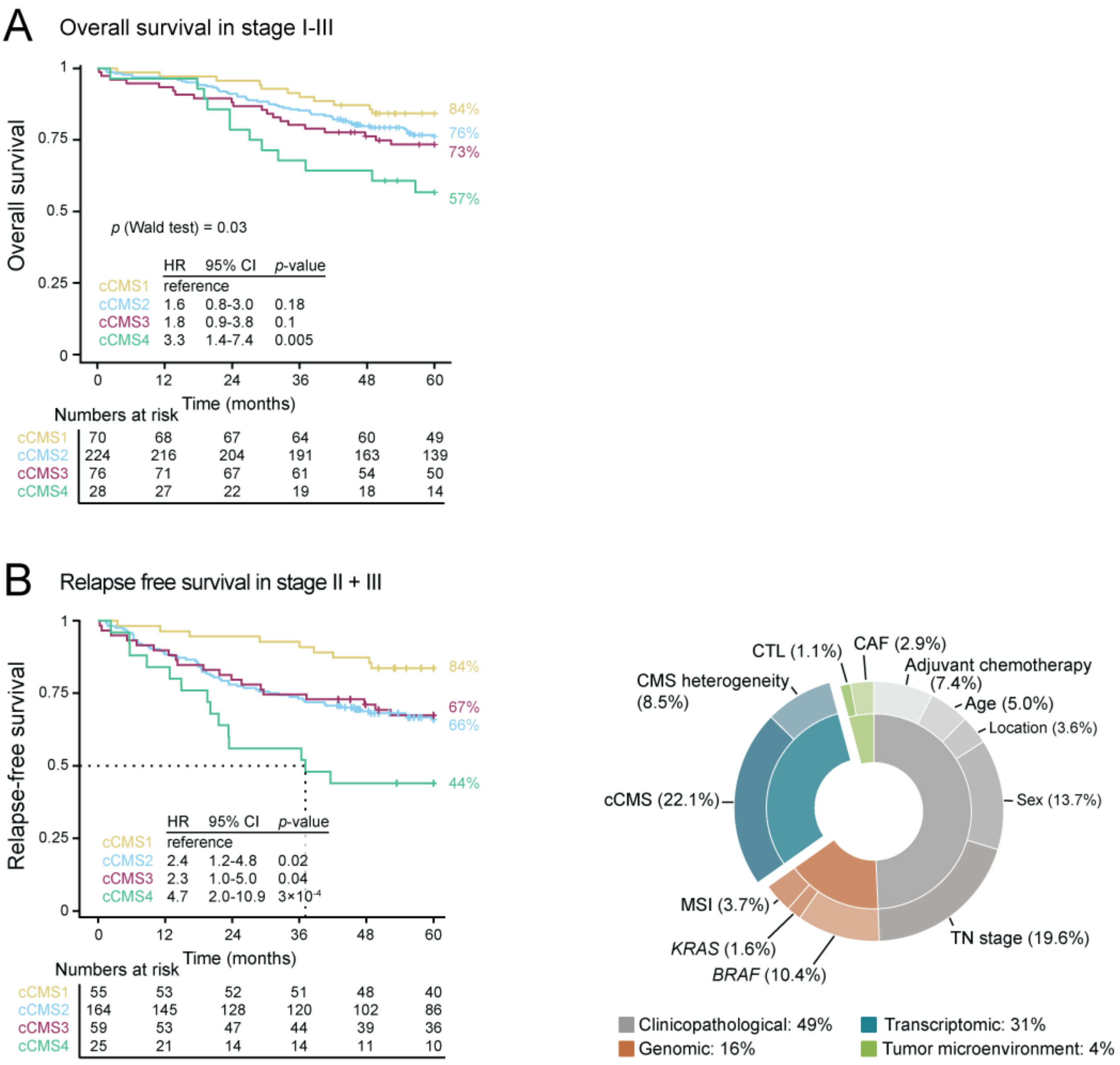

Supplementary Figure 27. Patient survival according to cCMS

A) Kaplan-Meier plot of five-year overall survival in patients with stage I-III CRC (*n* = 398 patients). B) Kaplan-Meier plot of five-year relapse-free survival in stages II and III CRC (left; *n* = 303 patients) and proportion of explained variation in survival by each variable in a multivariable Cox proportional hazards model, estimated as the percentage of the full model (right). Patients with incomplete tumor resection (R-status ≠ 0), heterogeneous intra-tumor cCMS classifications, synchronous tumors, or pre-surgical radiation treatment were excluded from analysis. Hazard ratios (HR) and 95% confidence intervals (CI) are from Cox proportional hazards analyses and *p*-values from Wald tests.

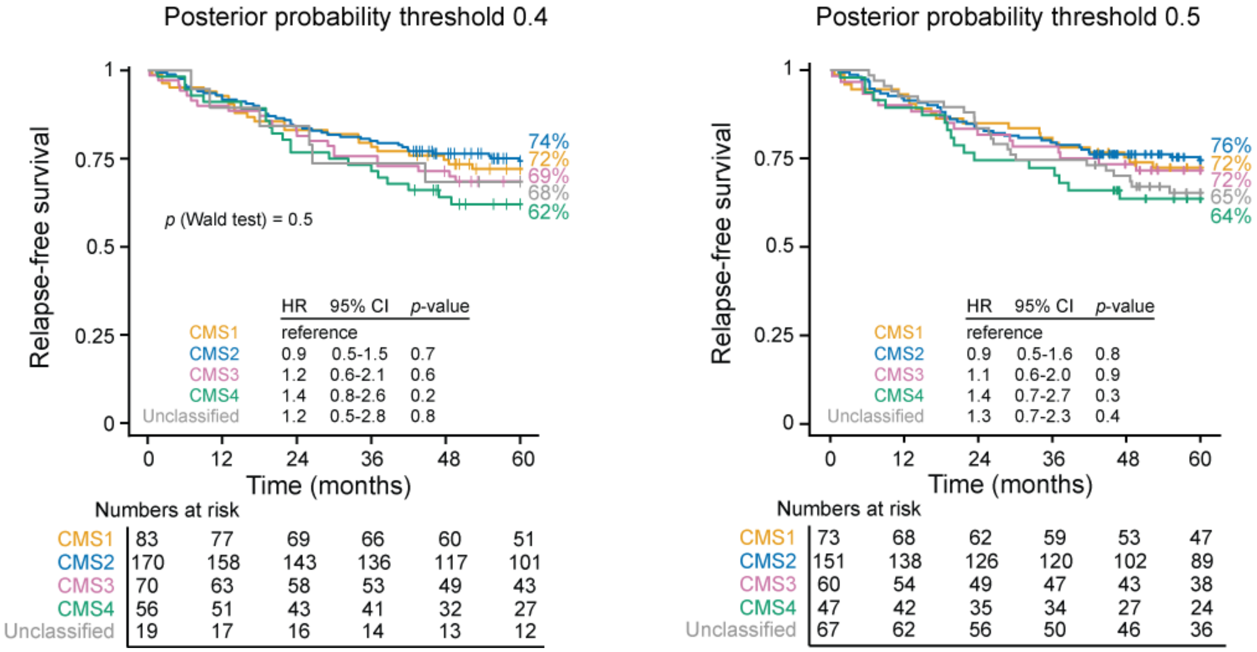

Supplementary Figure 28. Kaplan-Meier plot of five-year relapse-free survival according to CMS in stage I-III CRC (in the subgroup of patients with concordant intra-tumor cCMS classifications)

Survival analysis according to CMS classification performed in the patient group analyzed in Figure 5d and 5e (patients with concordant intra-tumor cCMS classifications, *n* = 398). Tumor samples were classified with the original CMSclassifier algorithm and a posterior probability threshold of 0.4 (left) or 0.5 (default; right). Hazard ratios (HR) and 95% confidence intervals (CI) are from Cox proportional hazards analyses and *p*-values from Wald tests.

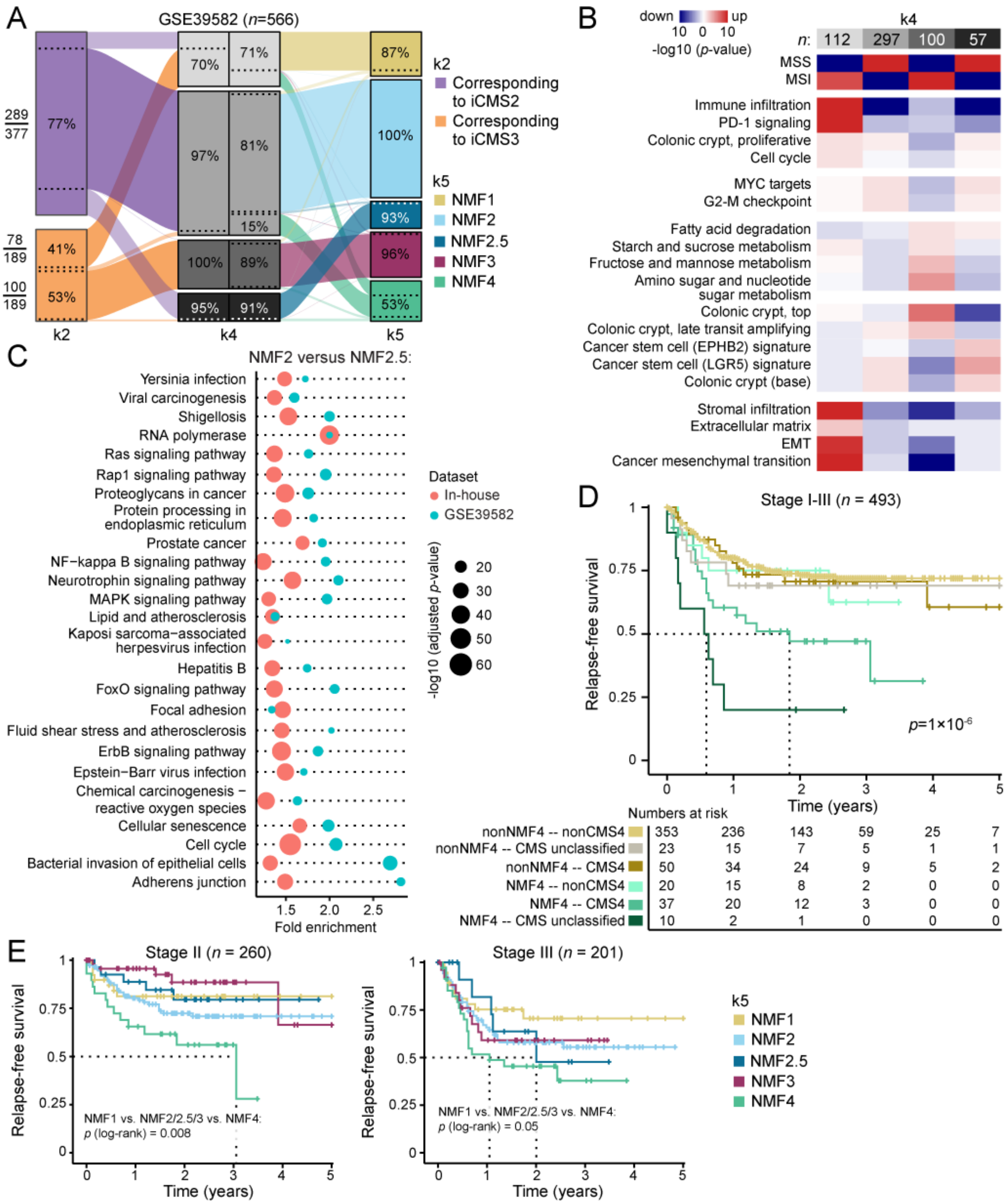

Supplementary Figure 29. Classification of the GSE39582 series (*n* = 566 primary tumors) based on ITH-low genes

A) Alluvial diagram of classification concordances between the ITH-low k2, k4 and k5 clusters from NMF. Sample overlaps are indicated relative to the total number per subtype. Source data are provided as a

Source Data file. B) Heat map of *p*-values (log10-scale) from enrichment analysis of the custom gene set collection according to the ITH-low k4 clusters (red: positive enrichments; blue: negative). Gene sets were selected to include the same as in the corresponding analysis of the in-house tumor series (Supplementary Figure 20). C) Common pathway enrichments for differentially expressed genes between the two CMS2-correspondent subtypes (NMF2 and NMF2.5) from k5 clustering of the in-house and GSE39582 series in the KEGG pathway database. All common pathways among the top-50 most significantly enriched in each dataset were included for plotting. Pathways are sorted alphabetically. Dot sizes indicate the significance level (Benjamini-Hochberg adjusted *p*-value on log10-scale). Source data are provided as a Source Data file. D) Five-year relapse-free survival of patients with stage I-III CRC grouped according to the original CMS4 class and the CMS4-correspondent class from ITH-low k5 clustering (class denoted NMF4). E) Relapse-free survival according to the k5 clusters in patients with stage II and III CRC separately. The *p*-values are from log-rank tests across subtypes.

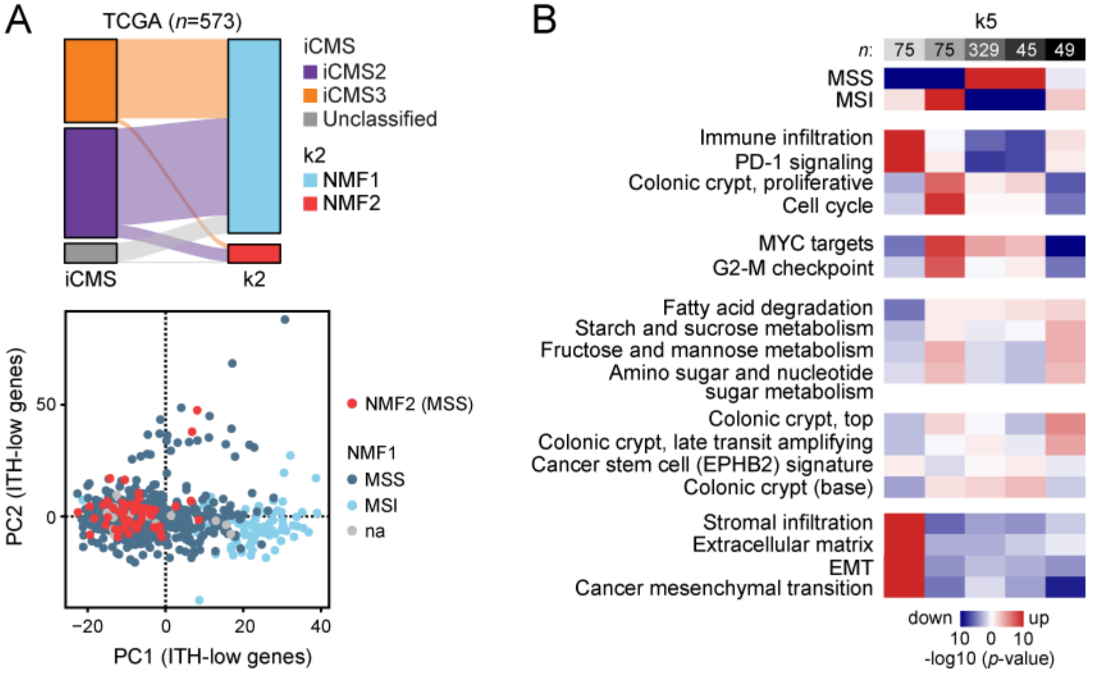

Supplementary Figure 30. Classification of the TCGA series (*n* = 573 primary tumors) based on ITH-low genes

A) Alluvial diagram of classification concordances between iCMS and the ITH-low k2 clusters (top), and principal components analysis based on ITH-low genes (bottom). The tumors were colored according to the k2 clusters and MSI status. Source data are provided as a Source Data file. B) Heat map of *p*-values (log10-scale) from enrichment analysis of the custom gene set collection according to ITH-low k5 clusters (red: positive enrichments; blue: negative). Source data are provided as a Source Data file.

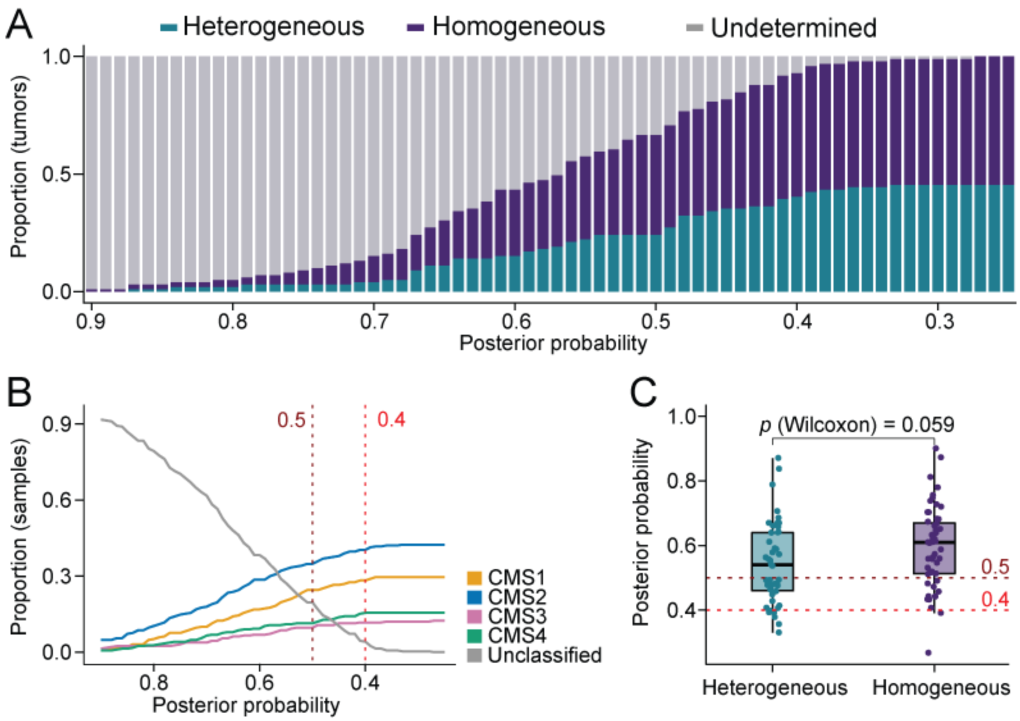

Supplementary Figure 31. The posterior probability threshold has a limited effect on the frequency of intra-tumor CMS heterogeneity

A) The proportion of tumors (*n* = 98) in the multiregional sample set (*n* = 286 samples) with heterogeneous and homogeneous CMS classification, as well as (B) the subtype proportions across the sample set, plotted according to posterior probability thresholds (intervals of 0.01) from the original random forest CMSclassifier. C) Box plot of the lowest posterior probability required to classify each tumor as either heterogeneous or homogeneous for CMS. Each data point represents one tumor, and tumors are grouped as homogeneous or heterogeneous for CMS at the indicated posterior probability. The posterior probability threshold for CMS classification did not have a strong impact on the relative proportions of tumors classified as homogeneous or heterogeneous, or on the relative distribution of the individual CMS classes. However, the proportion of unclassified samples and tumors varied inversely with the posterior probability, and appeared to stabilize at a low level at a posterior probability of approximately 0.4. The center line of boxes represents the median, boxes represent the interquartile range, and whiskers represent 1.5× the interquartile range above the 75th percentile (maxima) or below the 25th percentile (minima).

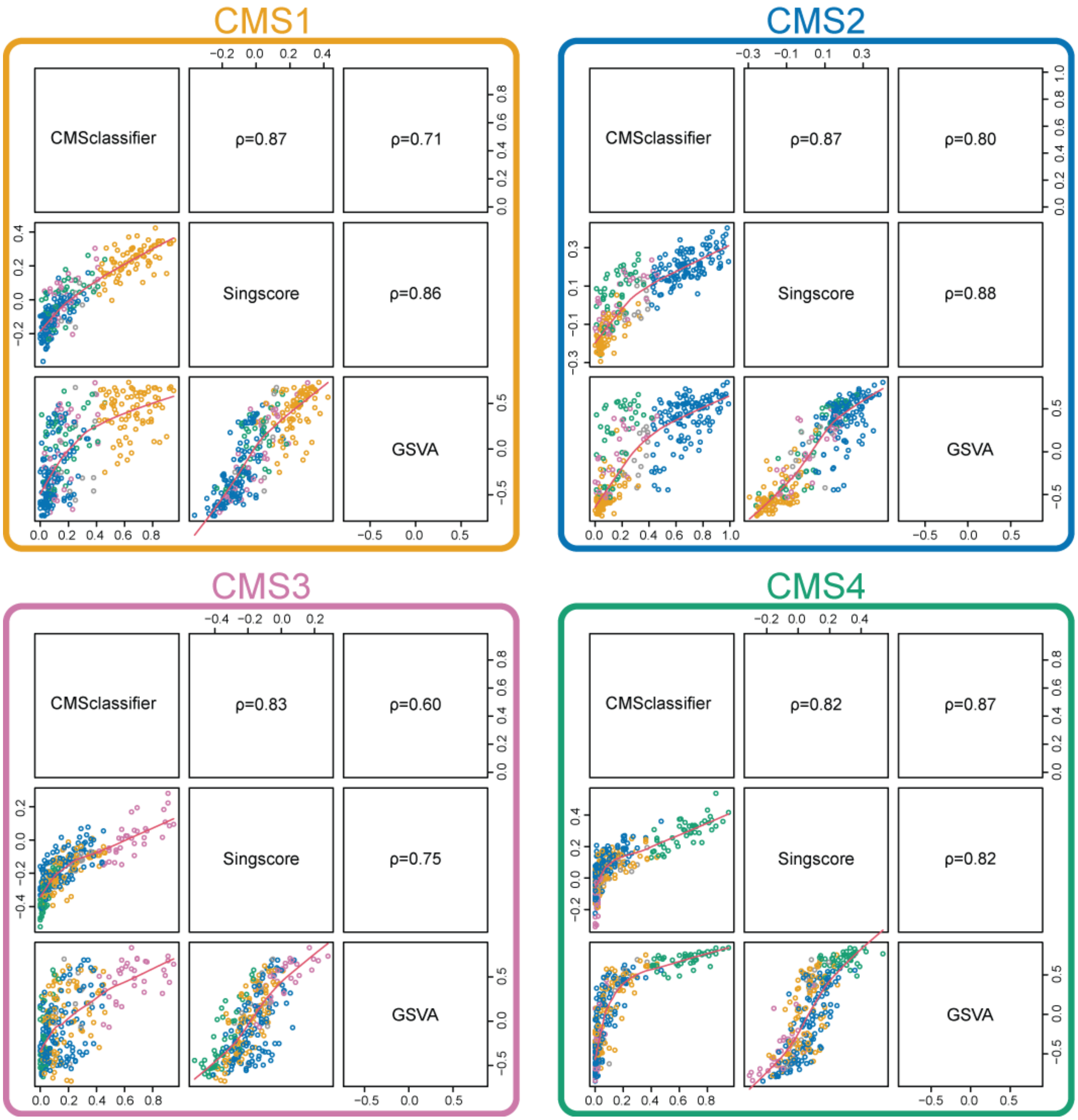

Supplementary Figure 32. Comparisons of CMS scores from different methods in the multiregional sample set

CMS scores for each sample (*n* = 286) were obtained as the posterior probabilities (threshold 0.4) from the original random forest CMSclassifier and as enrichment scores of CMS-specific gene sets using the GSVA and singscore algorithms (GSVA: based on up-regulated genes only). The lower left panels of each matrix show scatter plots of the respective CMS scores, with samples colored according to CMS class based on the CMSclassifier. The upper right panels show the corresponding Spearman's rank correlation coefficients (ρ).

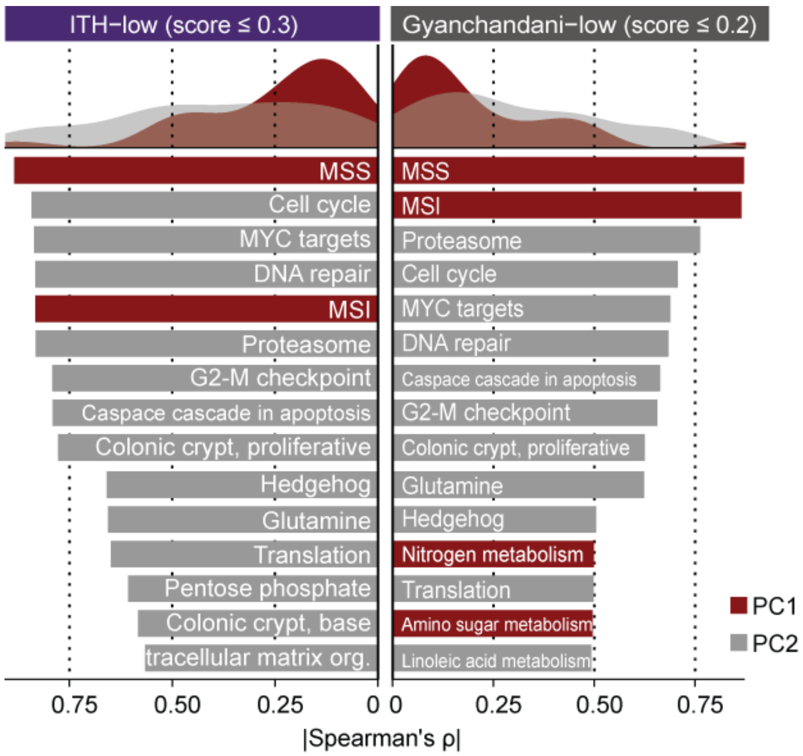

Supplementary Figure 33. Gene set correlations for ITH-low genes according to original and custom ITH-score thresholds

Left and right plots compare results for ITH-low genes defined according to custom and previously published ITH-score thresholds (Gyanchandani et al. Clin Cancer Res 2016;22:5362-9), respectively. The density plots show Spearman's correlation coefficient (ρ; absolute values) from correlation analyses of principal components (PCs) 1 (red) or 2 (grey) of ITH-low genes with GSVA scores of the custom gene set collection (n = 54) in the multiregional sample set (n = 286 samples). The barplots show the 15 top ranked gene sets from the corresponding analyses (ranked according to the correlation coefficient).
