# Supplementary material for: Multiregional transcriptomics identifies congruent consensus subtypes with prognostic value beyond tumor heterogeneity of colorectal cancer
Source: Nat Commun. 2024 May 21;15:4342. doi: 10.1038/s41467-024-48706-2 (PMC11109119; doi:10.1038/s41467-024-48706-2)
Supplement: Supplementary file 4 — Description of Additional Supplementary Files [file 41467_2024_48706_MOESM4_ESM.pdf]

Description of Additional Supplementary Files

File Name: Supplementary Data 1

Description: Clinicopathological and molecular characteristics of the primary CRC series

File Name: Supplementary Data 2

Description: Enrichment analyses of a custom gene set collection (n = 54) comparing heterogeneous and homogeneous tumors according to CMS in the multiregional sample set

File Name: Supplementary Data 3

Description: Enrichment analysis of a custom gene set collection (n = 54) comparing heterogeneous and homogeneous tumors according to each of the CMS classes in the multiregional sample set

File Name: Supplementary Data 4

Description: Differentially expressed genes (FDR < 0.05; n = 1,342) between tumors with heterogeneous and homogeneous CMS classifications in the multiregional sample set

File Name: Supplementary Data 5

Description: Clinical and molecular associations of CMS heterogeneity in the multiregional and extended sample series

File Name: Supplementary Data 6

Description: Univariable and multivariable survival analysis of patients with stage I-III CRC according to intra-tumor CMS heterogeneity

File Name: Supplementary Data 7

Description: Summary of CMS and CRIS combinations in the multiregional primary tumor sample set (n = 98 tumors)

File Name: Supplementary Data 8

Description: Summary of genes that passed and failed filtering based on inter-tumor expression variation (10-90th percentile range > 1), grouped according to ITH-score (custom thresholds)

File Name: Supplementary Data 9

Description: ITH-scores and categories of genes (according to custom thresholds)

File Name: Supplementary Data 10

Description: Clinicopathological and molecular associations of the CMS and cCMS classification frameworks

File Name: Supplementary Data 11

Description: Multivariable survival analysis of patients with stage I-III CRC according to cCMS classification

File Name: Supplementary Data 12

Description: Clinicopathological and molecular associations of k5 and k6 classifications of two external datasets (GSE39582 and TCGA) based on ITH-low genes

File Name: Supplementary Data 13

Description: Differentially expressed genes in each CMS class in the single-sample primary tumor set (samples analyzed on HTA; n = 217)

File Name: Supplementary Data 14

Description: Differentially expressed genes in each CMS class in the single-sample primary tumor set (samples analyzed on HuEx; n = 201)
